# Targeting Myeloperoxidase Ameliorates Gouty Arthritis: A Virtual Screening Success Story

**DOI:** 10.1021/acs.jmedchem.4c00721

**Published:** 2024-07-11

**Authors:** Isaac de A. Matos, Jorge L. Dallazen, Lorenna R. Reis, Luiz Felipe Souza, Regina C. Bevevino, Rafael D. de Moura, Graziella E. Ronsein, Nicolas Carlos Hoch, Nivan Bezerra da Costa Júnior, Soraia Kátia P. Costa, Flavia C. Meotti

**Affiliations:** † Department of Biochemistry, Institute of Chemistry, University of São Paulo, São Paulo 05508-000, Brazil; ‡ Department of Pharmacology, Institute of Biological Sciences, University of São Paulo, São Paulo 05508-000, Brazil; § Department of Chemistry, Federal University of Sergipe, São Cristóvão 49100-000, Sergipe, Brazil

## Abstract

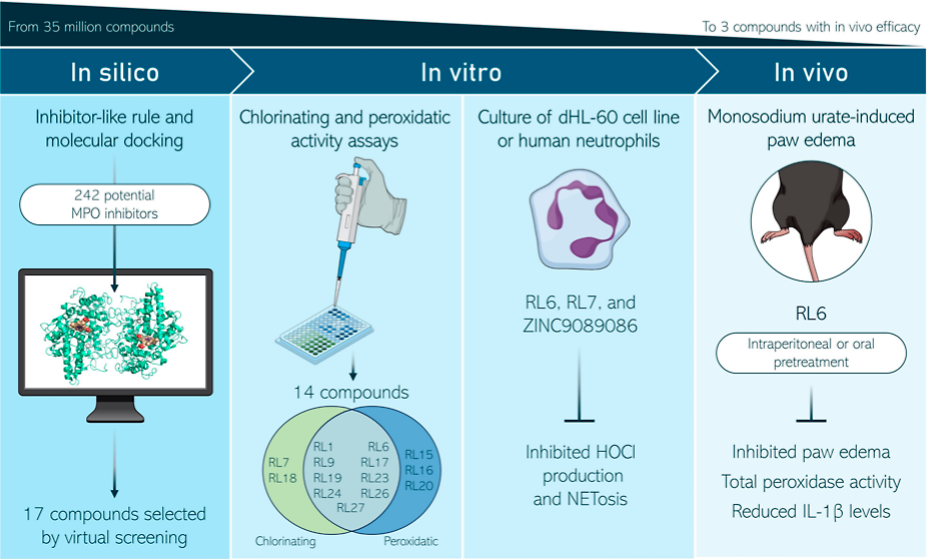

This study presents a new approach for identifying myeloperoxidase (MPO) inhibitors with strong in vivo efficacy. By combining inhibitor-like rules and structure-based virtual screening, the pipeline achieved a 70% success rate in discovering diverse, nanomolar-potency reversible inhibitors and hypochlorous acid (HOCl) scavengers. Mechanistic analysis identified RL6 as a genuine MPO inhibitor and RL7 as a potent HOCl scavenger. Both compounds effectively suppressed HOCl production in cells and neutrophils, with RL6 showing a superior inhibition of neutrophil extracellular trap release (NETosis). In a gout arthritis mouse model, intraperitoneal RL6 administration reduced edema, peroxidase activity, and IL-1β levels. RL6 also exhibited oral bioavailability, significantly reducing paw edema when administered orally. This study highlights the efficacy of integrating diverse screening methods to enhance virtual screening success, validating the anti-inflammatory potential of potent inhibitors, and advancing the MPO inhibitor research.

## Introduction

**Figure 1:**
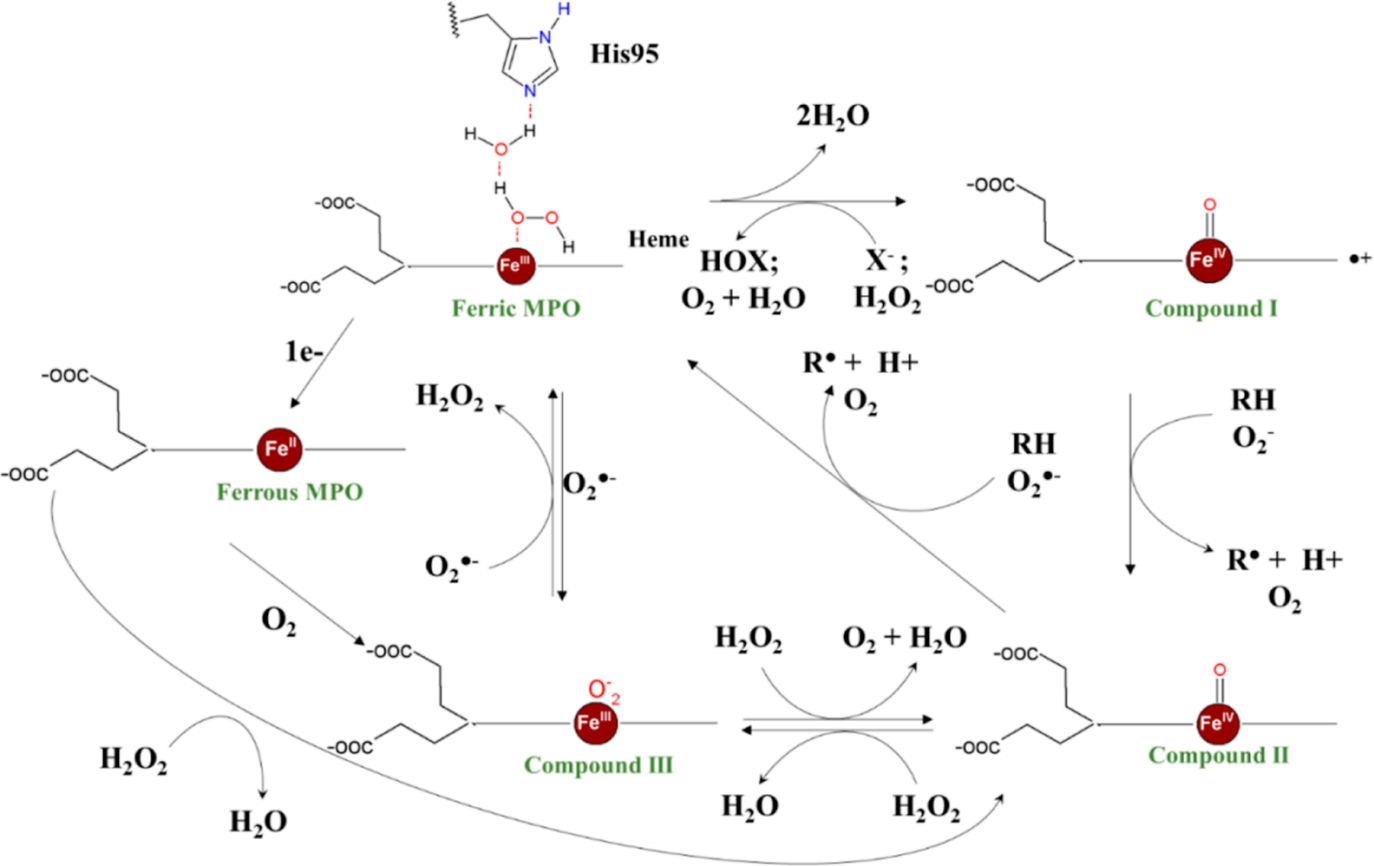
MPO catalytic mechanism. HOX-hypohalous acid, X^–^-halide ion, RH-oxidable. RH-includes alternative substrates such as urate.

According to a recent analysis of the Global Burden of Disease (GBD, 2019), approximately 1.71 billion people worldwide are living with musculoskeletal inflammatory conditions, such as rheumatoid arthritis, gout, neck pain, fractures, and other injuries.^1^ The primary responder cells, namely macrophages and neutrophils, appear early during inflammation and aggravate bone tissue damage, leading to bone erosion in the joints. Thus, they play a crucial role in the progression of inflammation.^2^ MPO, EC 1.11.2.2, is one of the most abundant proteins in neutrophil granules, known to be important in inflammation and immune defense.^3^ It is a homodimeric glycosylated enzyme belonging to the mammalian peroxidase superfamily, along with thyroid peroxidase (TPO), eosinophil peroxidase (EPO), and lactoperoxidase (LPO),^4^ being predominantly expressed in neutrophils^5^ but also found in monocytes, macrophages, and glial cells.^3,6,7^ Although MPO is important for controlling infection, it has been associated with tissue damage and chronic inflammation, contributing to the pathophysiology of various diseases,^8^ including Parkinson disease,^9^ cancer,^10^ multiple sclerosis,^11^ autoimmune diseases,^12^ atherosclerosis, and myocardial infarction.^7,13^ It catalyzes the oxidation of chloride by hydrogen peroxide, generating HOCl, a potent oxidant with microbicide activity.^14^ In this catalysis, the native ferric MPO (Fe^III^) reacts with hydrogen peroxide generating the intermediate compound I (Fe^IV^=O, porphyrin radical) (Figure 1).^14^ Compound I can oxidize halides and pseudohalides, yielding the corresponding hypohalous/pseudohypohalous acid and the native enzyme in a halogenation (chlorination) cycle. Alternatively, compound I can abstract one electron from organic substrates such as urate and tyrosine to form compound II (Fe^IV^=O) and the corresponding organic free-radical products (Figure 1). The abstraction of a second electron from these substrates completes the turnover in a peroxidatic cycle.^14,15^

In addition to HOCl production, MPO is involved in cytokine release^3^ and NETosis,^16^ a distinct type of neutrophil death characterized by the release of chromatin coated with cytotoxic proteins, capable of neutralizing microorganisms.^17^ While NETosis is crucial for killing microorganisms, it can also contribute to the pathophysiology of certain diseases.^18^ For instance, neutrophil extracellular traps (NETs) were found to be elevated in the plasma, tracheal aspirate, and lungs of Covid-19 patients, and their release by SARS-CoV-2-activated neutrophils caused lung epithelial cell death in vitro.^19^ NETosis has also been described in gouty arthritis, an inflammatory disease caused by uric acid deposition in the joins that affects approximately 7% of the world’s population. NETs is a key mechanism for clearance and inflammation resolution in gout.^20^ NETosis-deficient mice exhibited exacerbated and chronic inflammation after monosodium urate (MSU) crystal injection.^21^ Conversely, another study revealed that neutrophil activation in MSU-induced gout did not interfere with resolution but led to an increase in MPO activity in the paws of mice.^22^

Despite their important role in the pathogenesis of inflammatory disorders, studies testing the efficacy of MPO inhibitors against gout are scarce. Our group has extensively demonstrated the pathophysiological role of uric acid as an alternative substrate for MPO. Although oxidation of uric acid by MPO can generate allantoin, a soluble product that can decrease crystal formation, the intermediates of this oxidation, uric acid free radical, urate hydroperoxide, and hydroxyisourate,^15,23,24^ cause oxidative imbalance,^24^ reduce the capacity to kill microorganisms,^25^ and impair endothelial cell function,^26^ all events that might hamper inflammation resolution. Altogether, these findings suggest that MPO is a promising molecular target for the development of new anti-inflammatory agents and gout arthritis therapy. Several approaches have been employed in the discovery of MPO inhibitors, including virtual screening methods such as molecular docking and pharmacophoric models as well as high-throughput screening.^27−30^ However, these methodologies have not yielded a combination of a high hit rate, chemical diversity, and in vivo efficacy. To date, there are no MPO inhibitors approved for clinical use, although thioxanthines have entered clinical trials (NCT05492877). Recently, we reported a virtual screening methodology for MPO inhibitors that integrated an inhibitor-like rule and molecular docking. The combination of these methods led to a high hit ratio and the identification of chemically diverse molecules.^31^

The inhibitor-like rule was developed using descriptors traditionally used to predict the permeability/bioavailability of molecules. The rule states that MPO inhibition and the respective bioavailability of inhibitors are more likely for molecules that meet the following criteria: (1) molecular mass between 174 and 396 Da; (2) ACD/logP between 0.1 and 4.37; (3) hydrogen-bond donors up to 7 and hydrogen bond receptors between 2 and 9; (4) rotatable bond count up to 9; and (5) topological surface area (TPSA) between 18 and 122 Å^2^. The inhibitor-like rule was applied to 35 million compounds from the Zinc 12 database (http://zinc.docking.org), resulting in the filtering of 6546 potential ligands. Subsequently, we validated a molecular docking protocol and applied it to the 6546 molecules. Molecular docking simulations, in conjunction with visual inspection, narrowed the list of putative ligands to 242. These were then docked to MPO using a validated protocol using AutoDock and visual inspection, leading to the selection of 10 compounds for testing against MPO activity. Among them, six compounds inhibited enzyme activity, resulting in 60% success rate of this strategy.^31^

In this study, we reanalyzed the set of 242 computational hits to select a new set of MPO inhibitors. Before selecting compounds, the molecular docking protocol was thoroughly reviewed using newly deposited MPO structures. Seventeen potential MPO inhibitors were chosen and tested in a comprehensive pipeline, which includes enzymatic and cellular experiments as well as a murine model of gouty arthritis. In line with the methodology’s robustness, more than 60% of the selected compounds inhibited the MPO chlorinating and peroxidatic activities. Two of the three tested compounds inhibited HOCl production and NETosis by neutrophils and HL-60 cells. Intraperitoneal and oral administrations of these two compounds inhibited paw edema in a murine model of MSU crystal-induced arthritis. In summary, integration of two different screening methodologies filtered chemically diverse compounds with a high success rate for inhibiting MPO. The tested compounds demonstrated efficacy and bioavailability in a gouty arthritis mouse model, indicating therapeutic potential.

## Results

### Revalidation of the Molecular Docking Protocol

A new virtual screening of the previously identified 242 computational hits that met the MPO inhibitor-like rule and exhibited a favorable binding mode in molecular docking was carried out.^31^ Initially, we conducted a comprehensive re-evaluation of the molecular docking protocol. Alignments of the newly deposited MPO structures (PDB 5QJ2, 5QJ3, 6WXZ, 6WY0, 6WY5, 6WY7, 6WYD, 7LAE, 7LAG, 7LAL, and 7LAN) revealed a limited presence of conserved water molecules, as illustrated in Figure S1A. Water molecules were primarily situated at the periphery of the catalytic pocket, except for the catalytic water molecules located under the hemic iron atom. This particular water cluster had the potential to obstruct ligands that aimed to coordinate with the iron atom. Consequently, all water molecules were removed prior to the commencement of the molecular docking simulations.

Furthermore, we reanalyzed the flexibility of the MPO active site through alignment. Consistent with our prior findings, visual inspection indicated that the residues within the MPO active site were predominantly rigid, with the most flexible residues being Asp218, followed by Glu116, Met411, and Glu102 (Figure S1B).^31^ However, their positioning on the exterior of the binding pocket suggests diminished relevance to the binding of small molecules. This flexibility may be attributed to solvent turbulence rather than induced fitting, and therefore, MPO was treated as a rigid entity during our molecular docking simulations. To further validate our molecular docking protocol, we performed cross-docking due to the availability of a substantial number of new cocrystallized MPO ligands. In this process, we simulated the ligands cocrystallized in PDB 7LAG and 7LAN within the MPO active site, employing the PDB 1CXP structure as a reference. The results of the cross-docking simulation indicated that the molecular docking parameters had been fine-tuned to accurately reproduce the experimental conformation of the MPO inhibitor. This was evident from the alignment of the simulated (green) and experimental (yellow) conformations of the two cocrystallized ligands (Figure S1C,D). In line with these findings, the reference root-mean-square (refRMS) values were calculated to be 1.07 and 0.81 Å^2^ for the ligands in PDB 7LAG and 7LAN, respectively. Furthermore, the histogram profile exhibited a clean distribution, confirming the validity of the molecular docking protocol.^32^

### Identification of New MPO Inhibitors

After revalidating the molecular docking protocol, we reanalyzed the binding mode of the sublibrary consisting of 242 potential MPO inhibitors.^31^ This analysis considered several parameters, including chemical diversity, uniqueness, binding energy, the number of hydrogen bonds, π-stacking interaction, and conformational histogram profiles.^31^ To prevent an overestimation of the hit rate, we excluded molecules that were analogous to those previously reported for their inhibitory activity. Subsequently, 17 high-scoring putative MPO inhibitors were purchased. Eleven compounds inhibited the MPO chlorinating activity, with inhibition ranging from 18 to 92%. On the other hand, six compounds were found to be inactive, resulting in a 65% success rate (Table 1). While chlorinating activity is the primary physiological function of MPO, in vitro assays for this activity are susceptible to interference, as some compounds can scavenge hypochlorous acid or taurine chloramine.^33^ Therefore, we also evaluated all compounds against the MPO peroxidatic activity.

**Table 1:**
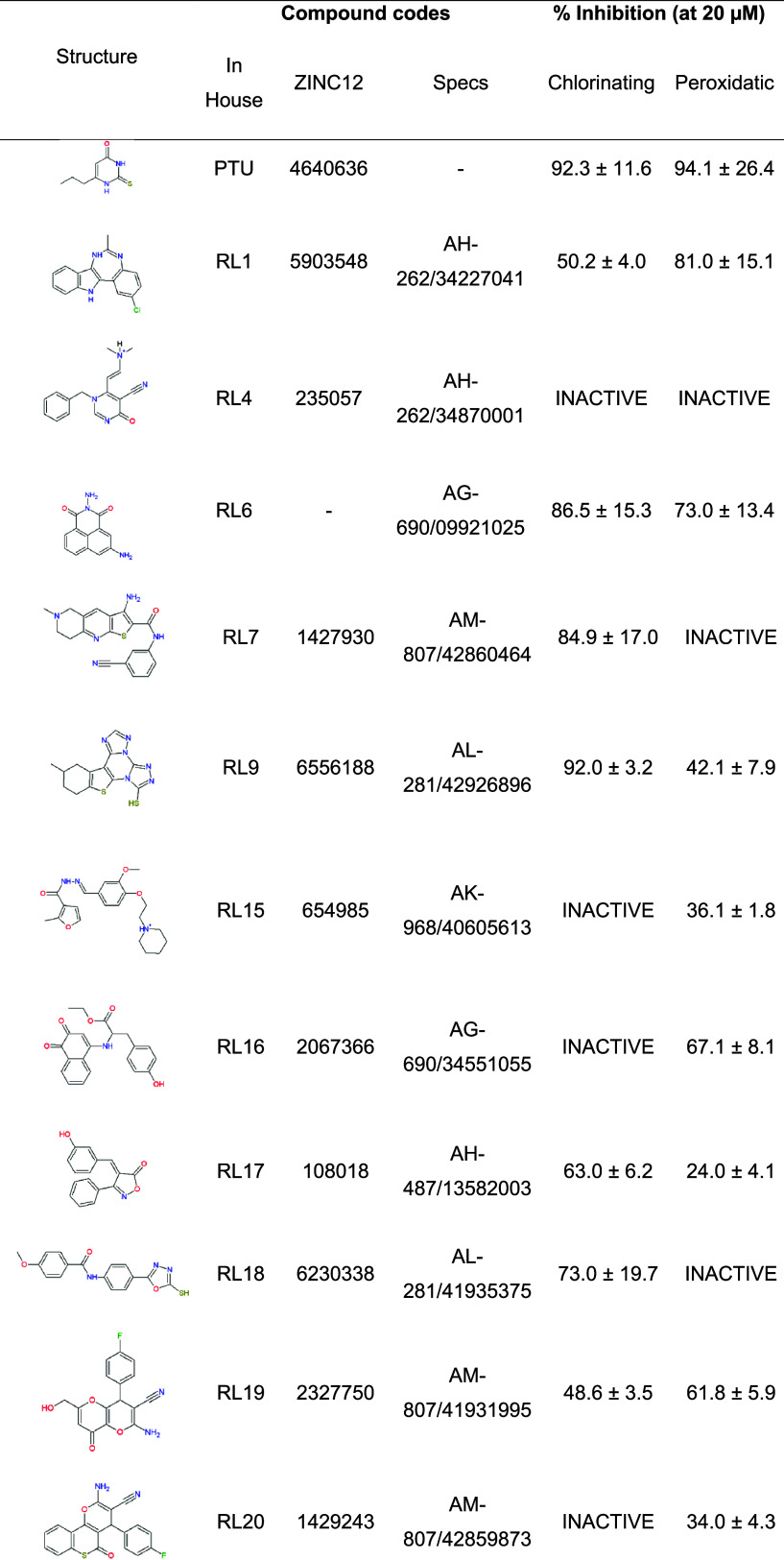

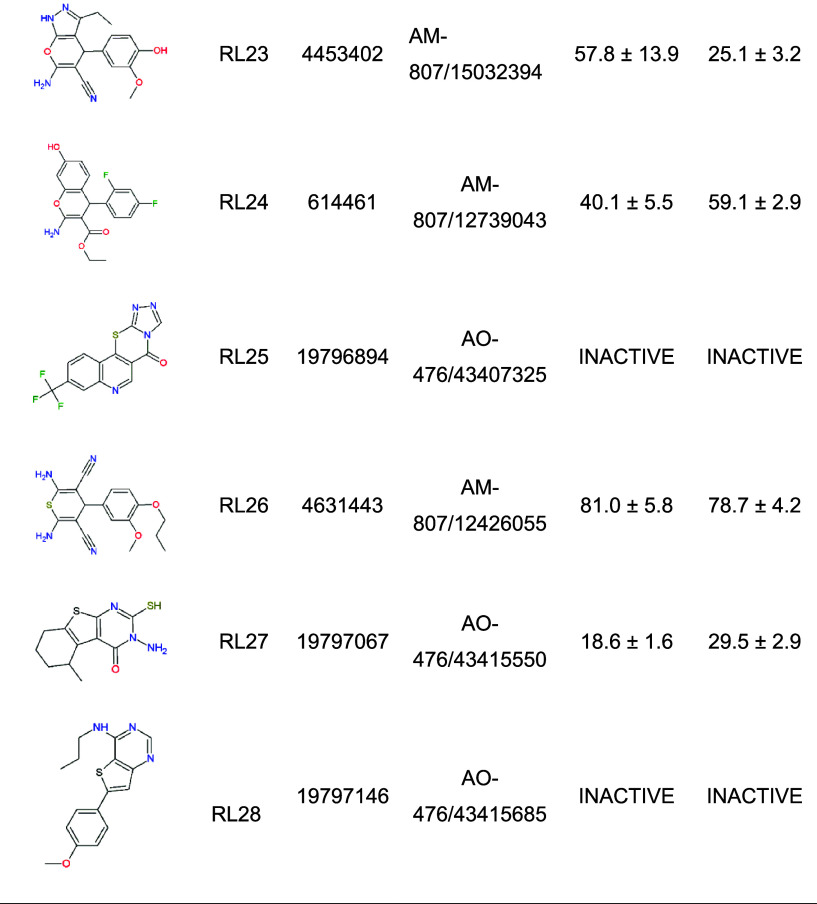
Inhibition of MPO Chlorinating and Peroxidatic Activities by Computational Hits

**Figure 2:**
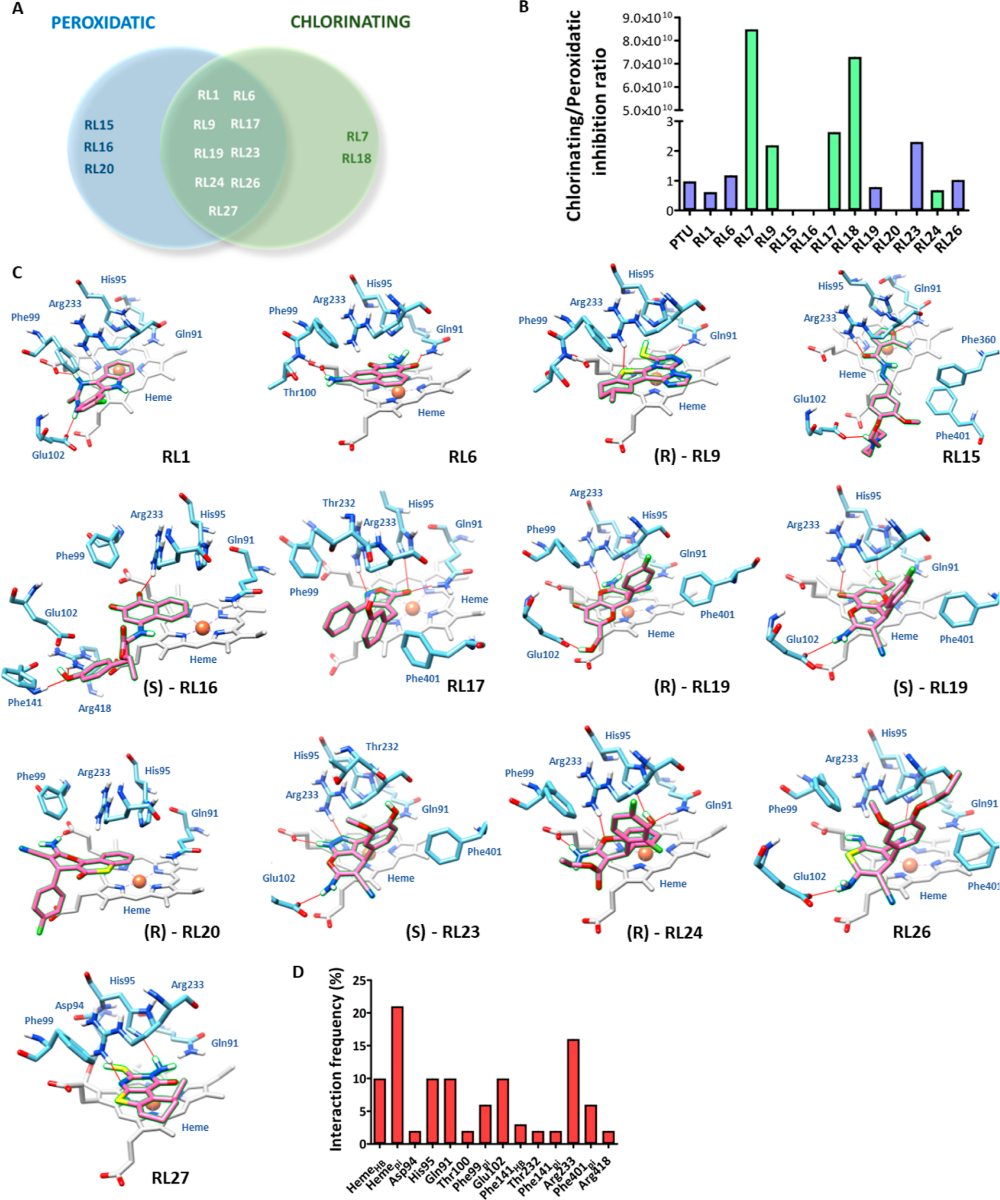
Inhibitors discovered by virtual screening and their binding modes from molecular docking. (A) Venn diagram showing the active compounds in the peroxidatic and chlorinating tests. (B) Chlorinating/peroxidatic activity inhibition ratio of the compounds. (C) Binding mode of the compounds showing the major interactions with the MPO active site. Residues are shown in blue, heme groups in gray, and ligands in pink. Hydrogen bonds and polar contacts are shown as red lines. For racemic compounds, the enantiomer is identified. (D) Frequency of interactions of the inhibitor-binding mode. Molecular docking was performed in PDB 1CXP protein structure using AutoDock 4.2.3.

In the peroxidatic assay, we measured MPO activity by monitoring AmplexRed oxidation,^34^ which is an artificial substrate but reduces scavenger interference. Twelve out of seventeen compounds inhibited peroxidase activity within a range of 24–81%. Only compounds RL4, RL7, RL18, RL25, and RL28 were inactive in this assay (Table 1), resulting in 70% success rate. Altogether, nine compounds inhibited MPO activity in both assays: RL1, RL6, RL9, RL17, RL19, RL23, RL24, RL26, and RL27. Three compounds, RL15, RL16, and RL20, inhibited exclusively the peroxidatic assay, while two compounds (RL7 and RL18) were active against chlorinating activity only (Table 1 and Figure 2A). This exclusive inhibition upon chlorination activity suggests that RL7 and RL18 are HOCl or taurine chloramine scavengers. The inactivities of RL15, RL16, and RL20 in the chlorinating assay may be attributed to the higher chloride concentrations used in the assay, which may increase stringency. The high chlorinating/peroxidatic activity ratio of RL9, RL17, and RL18 (Figure 2B) also suggests an important scavenger mechanism for these compounds. A chlorinating/peroxidatic activity ratio close to ∼1 indicates a true enzyme inhibitor (Figure 2B). Structural analysis of the active compounds reveals a significant number of novel chemical and pharmacological backbones, including benzodiazepine (RL1), naphthalimide (RL6), and azoles (RL9, RL18, RL23, and RL25) (Table 1).

### Active Compounds Exhibit Diversified Interaction Modes

Next, we analyzed the interactions of the active compounds with residues within the MPO active site (Figure 2C). The analysis of interaction frequencies indicates that the most common interaction is π stacking with the heme group (Figure 2D), representing 21% of the total interactions. This is followed by hydrogen bonds, which account for 16% of interactions involving Arg233, the heme group, His95, Gln91, and Glu102. Pi stacking with Phe99 and Phe401 represents 6%, while other interactions account for 2–3%, including Asp94, Thr100, Phr141_HB_, Thr232, Phe141_pi_, and Arg418.

RL1, the first benzodiazepine reported as an MPO inhibitor, forms two hydrogen bonds: one with Glu102 carboxylate and another with the heme group. Moreover, the indole group in RL1 is positioned beneath the heme plane, exhibiting a sandwich π stacking interaction with the heme π system. The benzene ring Choro-functionalized in RL1 also engages in a π stacking interaction but with the Phe99 residue in a T-shaped angle. The orientation of indole near the iron atom suggests that it could serve as a good substrate for MPO compound I, as the electrons from the indole ring can potentially be removed by the oxyferryl center, similar to what has been reported for tryptamines.^35^

RL6 shares structural similarity with 4-aminobenzoic acid hydrazide (ABAH), a classical irreversible MPO inhibitor.^36^ However, the RL6 chemical class, naphthalimide, has never been reported as an MPO inhibitor before. RL6 forms hydrogen bonds with Gln91 and the heme carboxylate in addition to a sandwich π stacking interaction between the tricyclic rings and the heme group. A T-shaped π stacking interaction is also likely between the naphthalene portion and Phe99. During complex dynamics, the hydrazyl group (H_2_N–N) putatively forms two additional hydrogen bonds with His95 (distal histidine), as proposed for hydrogen peroxide and hydroxamates.^37,38^ It also makes a hydrogen bond with Arg233, totaling five putative hydrogen bonds for this inhibitor. RL9 can be classified as a thiol poliazole that has a stereogenic center and tautomeric forms, represented by **R** and **S** enantiomers and by the thione and thiol tautomers (Tables 1 and S1). Molecular docking of this species indicates that the **R** enantiomer in thiol form has the most favorable binding free energy (Supporting Information Table S1). It interacts via two hydrogen bonds with the Gln91 and Arg233 residues. The large quadricyclic ring in this molecule forms sandwich stacking interactions with the heme π system and Phe99 (at a T-shaped angle) (Figure 2C). Interestingly, the thiol group is positioned beneath the hemic iron, as reported for propylthiouracil (PTU) binding to lactoperoxidase (PDB 5HPW). The hydrogen is orientated toward the tau nitrogen atom in His95, suggesting a putative hydrogen bond, similar to the binding mode reported for hydrogen peroxide and hydroxamates.^37,38^ RL15, a phenyl hydrazone, forms hydrogen bonds with Gln91, Arg233, and Glu102, the latter exhibiting a high electrostatic nature. The furan and benzene rings form π stacking interactions with the heme and potentially with Phe360, respectively. Inhibitor RL16 is an enantiomeric vicinal naphthalene dione, and the **S** enantiomer presents the best binding mode (Table S1). This species forms hydrogen bonds with Arg233, Phe141, and Arg418 residues, unlike other compounds (Figure 2C). Theoretically, the formation of a hydrogen bond with Glu102 is expected during solution dynamics. Moreover, π stacking interactions with the heme (sandwich) and Phe99 and Phe141 (T-shaped angle) were observed. Analysis of the oxazolone RL17 indicates an unfavorable contact with tau nitrogen in His95. Despite this repulsive contact, this compound forms hydrogen bonds with Arg233 and Gln91, as well as π stacking interactions between the oxazole ring and the heme plane. Surprisingly, two benzene rings in RL17 are parallel to each other and localized between residues Phe99 and Phe401, indicating a large contribution of sandwich π stacking to complex stabilization. Molecular docking of RL19, a chiral chromeno, indicates that both **R** and **S** enantiomers exhibit favorable binding parameters (Table S1). Binding energy values reveal a slight preference for the **R** enantiomer. This species forms hydrogen bonds with Glu102, Arg233, and His95. Besides, an extra bond with Gln91 could be formed with the nitrile group during the protein motion. Interestingly, the orientation of the amino group related to His95 is similar to the binding mode of the hydrazyl and thiol groups in RL6 and RL9, respectively (Figure 2C). Additionally, RL19 is stabilized by sandwich π stacking interactions between the chromeno and heme groups. The chromeno ring also interacts with the Phe99 residue, forming a T-shaped angle. In addition, a dislocated π stacking interaction can be formed between the fluorophenyl group and the Phe401 residue. The RL19 **S** enantiomer exhibits a similar binding mode to the **R** enantiomer, but the hydroxymethyl group is positioned inside the active site, forming a hydrogen bond with His95. The chromeno carbonyl group interacts with Arg233. Finally, the amino group in the **S** enantiomer forms a hydrogen bond with Glu102. Although the RL20 inhibitor is also a chiral chromeno derivative, molecular docking simulations indicate a different binding mode from the chromenos described above (Figure 2C), with the **R** enantiomer being the most likely conformation (Table S1). In comparison to RL19, the fluorophenyl ring is oriented oppositely, and RL20 forms only one hydrogen bond with the heme carboxylate and a sandwich π stacking interaction with the planar π system. RL23 exhibits a structure and binding mode similar to those of RL19. However, the former is a diazole and not a chromeno, and the phenyl ring contains a hydroxyl group instead of a fluor. The **S** enantiomer exhibits a more concerted conformation (Table S1), forming hydrogen bonds with Arg233, Glu102, and the heme carboxylate (Figure 2C). Pi stacking interactions are observed between the diazole ring and the heme π system as well as with Phe401 through the methoxyphenol portion. RL24 is a chiral chromeno derivative that bears significant analogy with RL19. The RL24 **R** enantiomer is the most favorable for interacting with the MPO active site (Table S1). Similar to RL19, RL24 also forms a hydrogen bond with His95, but the hydroxyl group is phenolic in the latter, whereas it is an alkyl hydroxyl in the former. This phenol hydroxyl forms a hydrogen bond with Gln91. RL24 also interacts through hydrogen bonds with Arg233 and the heme carboxylate by the oxygen heteroatom from the chromeno ring and by the amino group, respectively. A sandwich π stacking interaction is observed between the heme π system and the chromeno ring. RL26 is a tadpole-shaped molecule with a symmetrical thiopyran ring functionalized with two amino and nitrile groups. This molecule forms one hydrogen bond with Glu102 and, considering the motion in solution, can potentially form two other hydrogen bonds with heme carboxylates and Arg233. One symmetric nitrile group makes an unfavorable contact with the tau nitrogen in His95. Furthermore, the phenyl ring bonded to thiopyran exhibits a π stacking interaction with Phe401 in the T-shaped angle. A residual aromaticity of the thiopyran ring might also interact with the heme π system. The hydrophobicity of the propyl chain in RL26, which is accommodated in a pocket between Arg233 and Phe401, can contribute to complex stabilization. The last inhibitor, compound RL27, displays structural similarity with the known MPO inhibitors, thioxanthines.^39^ Despite this similarity, RL27 has the difference of a hydrazyl group and the thiofuran as the five-member rings. Furthermore, this molecule can be represented by **R** and **S** enantiomers and by a thiol/thione tautomer. Molecular docking studies indicate that the **S** enantiomer, i.e., the thiol tautomer form, exhibits the best binding energy to the MPO active site (Table 1). The inspection of the MPO–RL27 complex reveals the presence of hydrogen bonds with the Arg233 and His 95 residues. Similar to the RL6 compound, the N–NH_2_ group in RL27 is oriented in a manner reminiscent as seen for hydrogen peroxide and hydroxamates.^37,38^ The proximity between the thiol and carbonyl groups of Asp94 suggests the occurrence of a hydrogen bond during protein dynamics. Molecular docking of RL27 also suggests a sandwich π stacking interaction with the heme π group.

### RL6 is a Potent Reversible Inhibitor

**Figure 3:**
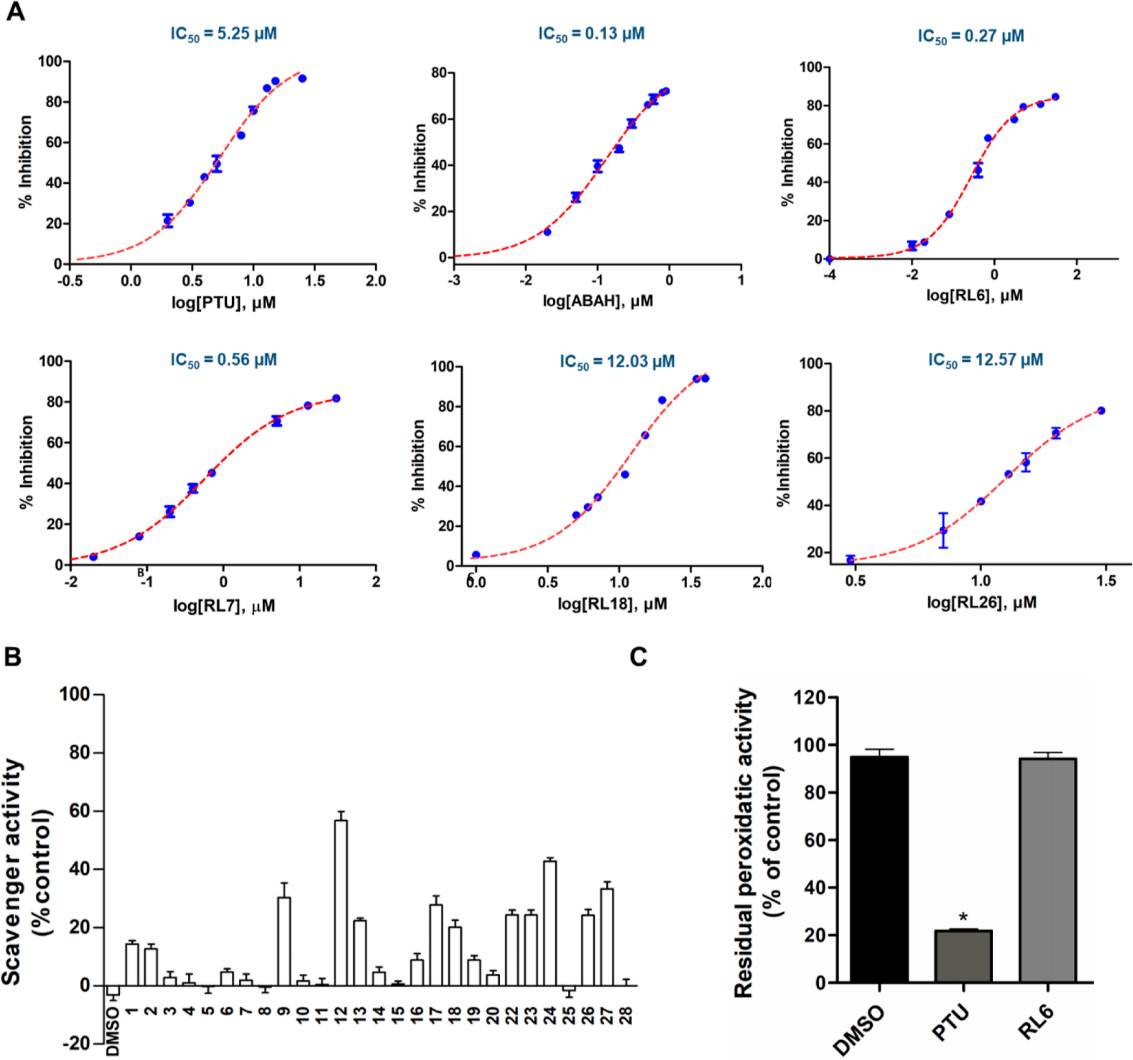
IC_50_ plots, scavenger activity, and residual activity of the compounds. (A) IC_50_ of the MPO chlorinating activity performed in 20 mM phosphate buffer, pH 7.4; compounds were dissolved in DMSO 0.03%, 10 nM MPO, 100 μM DTPA, 0.03% CTAC, 140 mM NaCl, and 5 mM taurine. Reaction was started with 40 μM hydrogen peroxide and stopped using catalase. Taurine chloramine was quantified by TMB oxidation. (B) Scavenger activity of the compounds toward taurine chloramine. For scavenger assay, 2.81 mM HOCl was mixed with 5 mM taurine in phosphate buffer (20 mM, pH 7.4, 140 mM NaCl, 100 μM DTPA), and after 5 min, the compounds were added to this mixture for 15 min at 37 °C. Taurine chloramine was quantified by the oxidation of TMB.^33^ (C) Residual peroxidase activity was carried out using 100 nM MPO with 20 μM inhibitors in phosphate buffer (20 mM, pH 7.4), 0.03% CTAB, and 40 μM H_2_O_2_ at 37 °C for 30 min. After incubation, the system was diluted 200-fold using acetate buffer (200 μM, pH 5.4), and the residual peroxidatic activity was detected by TMB. Bars represent mean ± SEM of three independent experiments (*n* = 3). Statistical analysis was performed using one-way ANOVA, followed by Bonferroni posthoc test. *statistically different (*p* < 0.01) compared to the control group (DMSO).

Next, we compared inhibitor potencies by plotting IC_50_ curves for those compounds that inhibited the chlorinating activity by approximately 70% at a concentration of 20 μM. RL6 and RL7 displayed IC_50_ values in the nanomolar range (Figure 3A). RL6 proved to be the most potent inhibitor, with an IC_50_ value of 270 nM. Interestingly, it did not scavenge taurine chloramine (Figure 3B), suggesting that it is an enzyme inhibitor. In support of this, RL6 exhibited a chlorinating/peroxidase activity ratio close to 1 (Figure 2B). The second most potent compound, RL7, had an IC_50_ value equal to 560 nM. Unlike RL6, RL7 might function as a direct HOCl scavenger, as it significantly inhibited the chlorinating activity while having a smaller impact on peroxidase activity (Figure 2B). However, it did not scavenge the milder oxidant taurine chloroamine (Figure 3B). RL26 had IC_50_ of 12.57 μM, likely attributed to both enzyme inhibition and scavenger activity, as it decreased both taurine chloroamine (Figure 3B) and MPO peroxidatic activity (Table 1 and Figure 3B). RL18 exhibited IC_50_ of 12.03 μM, primarily ascribed to its scavenger capacity (Figure 3B), as it did not inhibit the MPO peroxidatic activity (Table 1 and Figure 2B). To validate the dose–response curves and IC_50_ calculations, we employed two known MPO inhibitors: PTU and ABAH. The calculated IC_50_ values were 5.25 and 0.13 μM, respectively (Figure 3A), which closely align with the previously reported values of 3.38 and 0.30 μM, respectively.^40,41^

Notably, RL6 displayed reversible inhibition, as MPO activity was recovered upon sample dilution in contrast to PTU (Figure 3C). A reversible inhibition is often associated with lower toxicity, consequently, RL6 may be a more promising MPO inhibitor in vivo than PTU, as it boasts a lower IC_50_ and potentially fewer side effects. To gain deeper insights into the superior potency of RL6 as a reversible inhibitor compared to ABAH, an aromaticity study using DFT (density functional theory) was carried out to quantify the importance of aromaticity in enzyme binding. The calculated aromaticity index, harmonic oscillator model of aromaticity (HOMA),^42^ was 0.81 and 0.82 for each ring in RL6, the sum of the two rings being 1.63. This indicates that the naphthalimide ring in RL6 exhibits greater aromaticity than the single ring in ABAH (HOMA 0.9456), facilitating π stacking interactions with the heme π system and, thereby, contributing to enhanced potency of RL6.

### Mechanisms of RL7 and RL6 Inhibition

**Figure 4:**
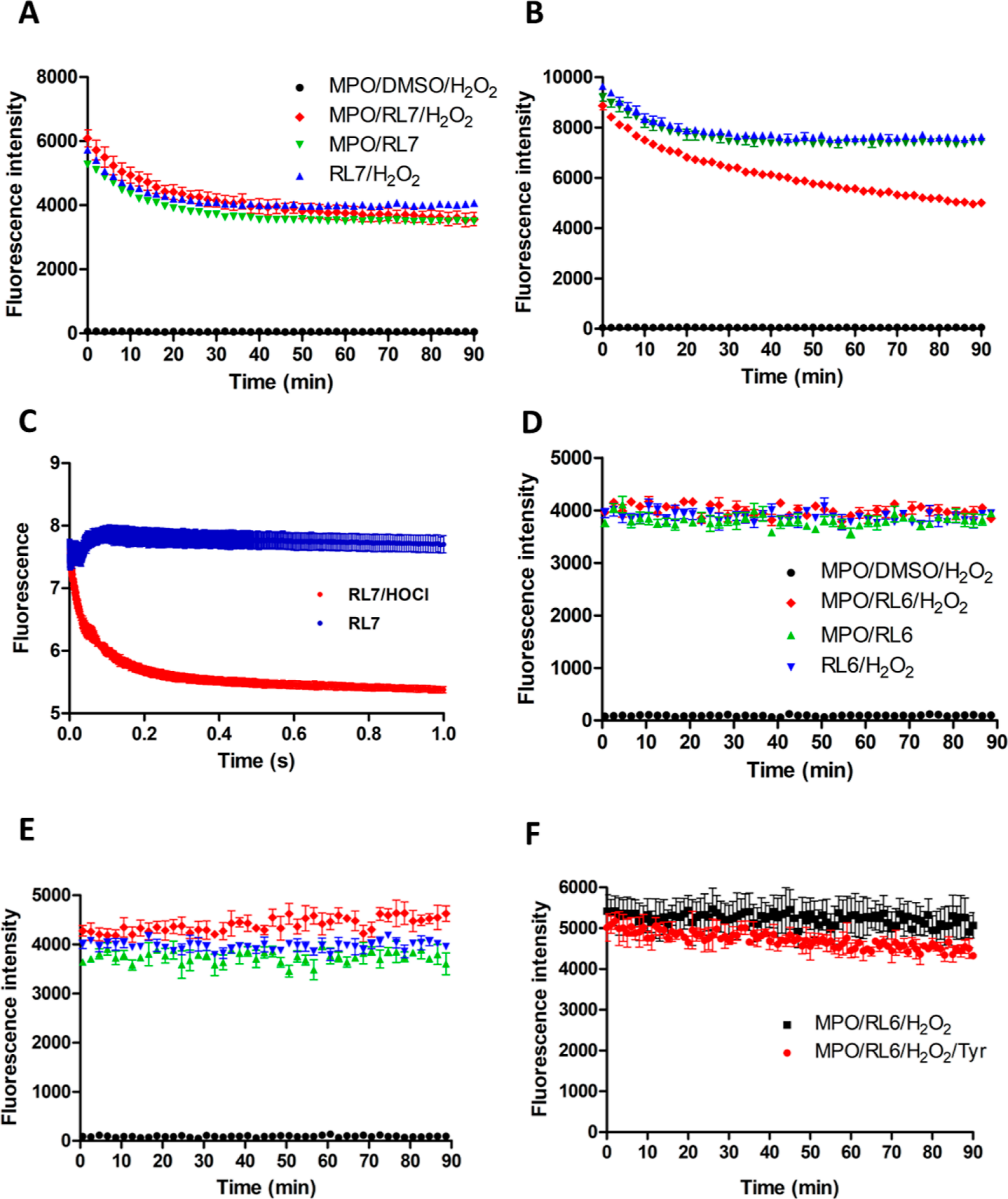
Evaluation of RL7 and RL6 oxidation by fluorescence decay. RL7 fluorescence after incubation with MPO/H_2_O_2_ in the absence (A) or presence (B) of chloride. Reactions were performed in phosphate buffer (20 mM, pH 7.4) containing 1 μM RL7 dissolved in DMSO (0.3% final concentration), 10 nM MPO, 100 μM DTPA, 5 mM taurine, 0.03% CTAC or CTA(SO_4_H^–^) for no chloride samples, and 140 mM NaCl. The reaction was started by adding 40 μM hydrogen peroxide and RL7 concentration monitored by fluorescence (λ_ex_ = 306/λ_em_ = 603 nm for RL7). (C) RL7 fluorescence decay in the absence (blue line) or presence (red line) of HOCl. Fluorescence of RL7 (20 μM in 0.3% DMSO) was monitored in a stopped-flow device using λ_ex_ = 306 nm and λ_em_ = above 340 nm in 20 mM phosphate buffer (pH 7.4) containing 100 μM DTPA and 140 mM. RL7 was rapidly mixed with 250 μM HOCl. The data were obtained up to 1 s after mixing. Fluorescence of RL6 after incubation with MPO/H_2_O_2_ in the absence (D) or presence (E) of chloride. Reaction was performed as for RL7. In (F) was added 50 μM tyrosine (Tyr). RL6 oxidation was monitored by λ_ex_ = 413/λ_em_ = 603 nm. Data represented mean ± SEM of three independent experiments (*n* = 3).

While additional information can be obtained from dose–response curves, such as the number of interaction sites, multiple ligand binding, and promiscuous inhibitor aggregators,^43,44^ Hill fit parameter analysis failed to distinguish between true inhibitors and scavenger agents (data not shown). Consequently, we next assessed the oxidation of RL6 and RL7 by MPO or HOCl through the analysis of their intrinsic fluorescence (Figure S2). Other constituents of the system MPO, DMSO, and hydrogen peroxide did not exhibit relevant fluorescence at the selected excitation/emission wavelengths. RL7 exhibited a slight spontaneous decrease in fluorescence, which was not further enhanced by hydrogen peroxide/MPO (Figure 4A). However, RL7 was further consumed in the full reaction system in the presence of chloride (Figure 4B). These data support the conclusion that RL7 is an HOCl scavenger, consistent with the high chlorinating/peroxidatic activity ratio (Table 1 and Figure 2B). To confirm this, we incubated 20 μM RL7 with 250 μM HOCl using a stopped flow to monitor fluorescence decay on a millisecond scale. An exponential decay upon incubation with HOCl was detected (Figure 4C), confirming that this compound rapidly reacts with HOCl.

**Figure 5:**
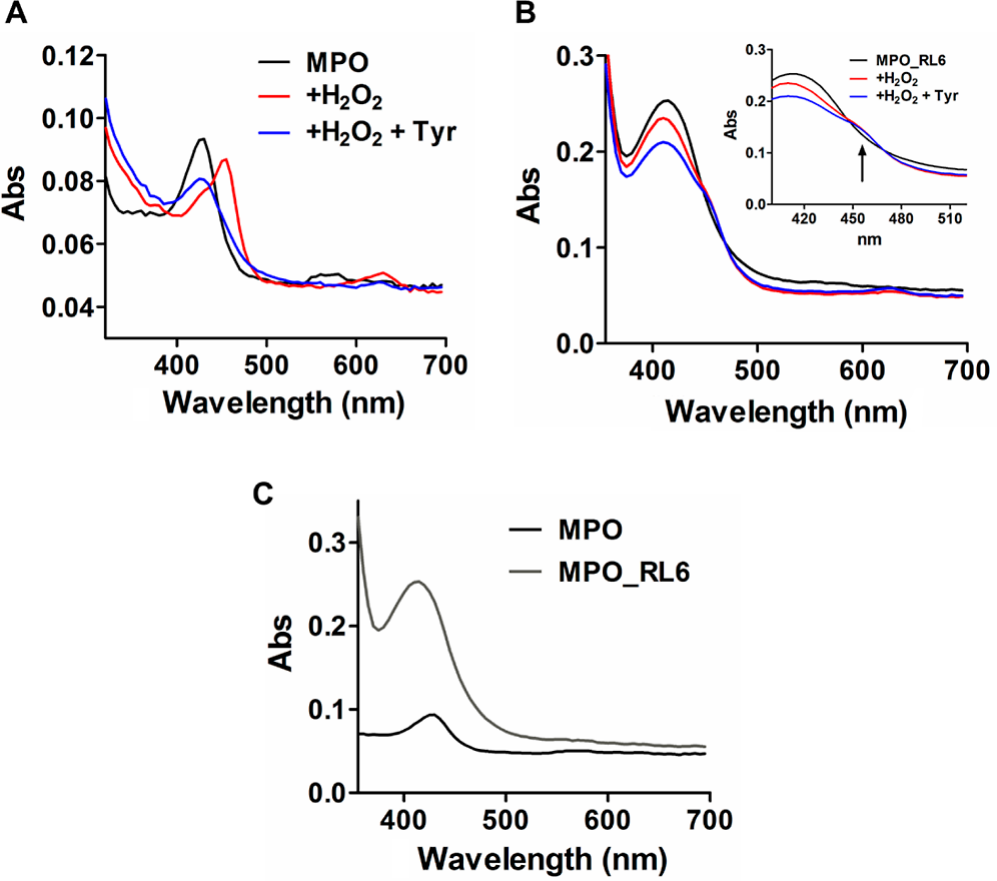
MPO spectra. MPO (0.5 μM) spectra were monitored in phosphate buffer (10 mM, pH 7.4) in (A) before (black line) and after reacting with 100 μM H_2_O_2_ (red line) and 100 μM H_2_O_2_ plus 50 μM tyrosine (blue line). (B) Same as in A, but in the presence of 10 μM RL6. Inset showing the stabilization of absorption at 456 nm even in the presence of tyrosine. (C) Comparison of the spectra of 0.5 μM MPO in the absence (black line) or presence (gray line) of 10 μM RL6.

In contrast to RL7, RL6 fluorescence did not undergo any alteration in the absence or presence of chloride, MPO, and hydrogen peroxide (Figure 4D,E). However, RL6 could potentially serve a substrate for MPO compound I, but not for compound II, which would trap the enzyme as compound II, preventing its complete turnover and the accumulation of oxidized RL6, similar to what occurs with tryptophan.^45^ To verify this possible mechanism, we tested the oxidation of RL6 by MPO/hydrogen peroxide in the presence of tyrosine, a well-described substrate for compound II that can re-establish the peroxydatic cycle,^46,47^ bypassing compound II accumulation. No oxidation occurred, even in the presence of tyrosine (Figure 4F), supporting that RL6’s inhibitory effect was not due to the trapping of compound II. MPO spectra in the presence of tyrosine further supported the absence of compound II accumulation by RL6 (Figure 5B). The addition of hydrogen peroxide caused a shift in the Soret peak of MPO from 430 to 456 nm and a slight increase in absorbance at 630 nm (Figure 5A black and red traces), indicative of conversion of the ferric enzyme to compound II (49). In the presence of tyrosine, the formation of compound II was transient, and the spectrum of the ferric enzyme predominated (Figure 5A blue trace). Even though RL6’s spectrum overlapped with that of MPO (Figure 5C, gray trace), it is possible to verify that the presence of tyrosine did not re-establish the ferric enzyme (Figure 5B, blue trace). RL6 was not oxidized in the presence of chloride, as well (Figure 4E), confirming that the compound does not function as a HOCl scavenger.

### MPO Inhibitors Decrease NETosis and HOCl Production in dHL-60 Cells and Neutrophils

**Figure 6:**
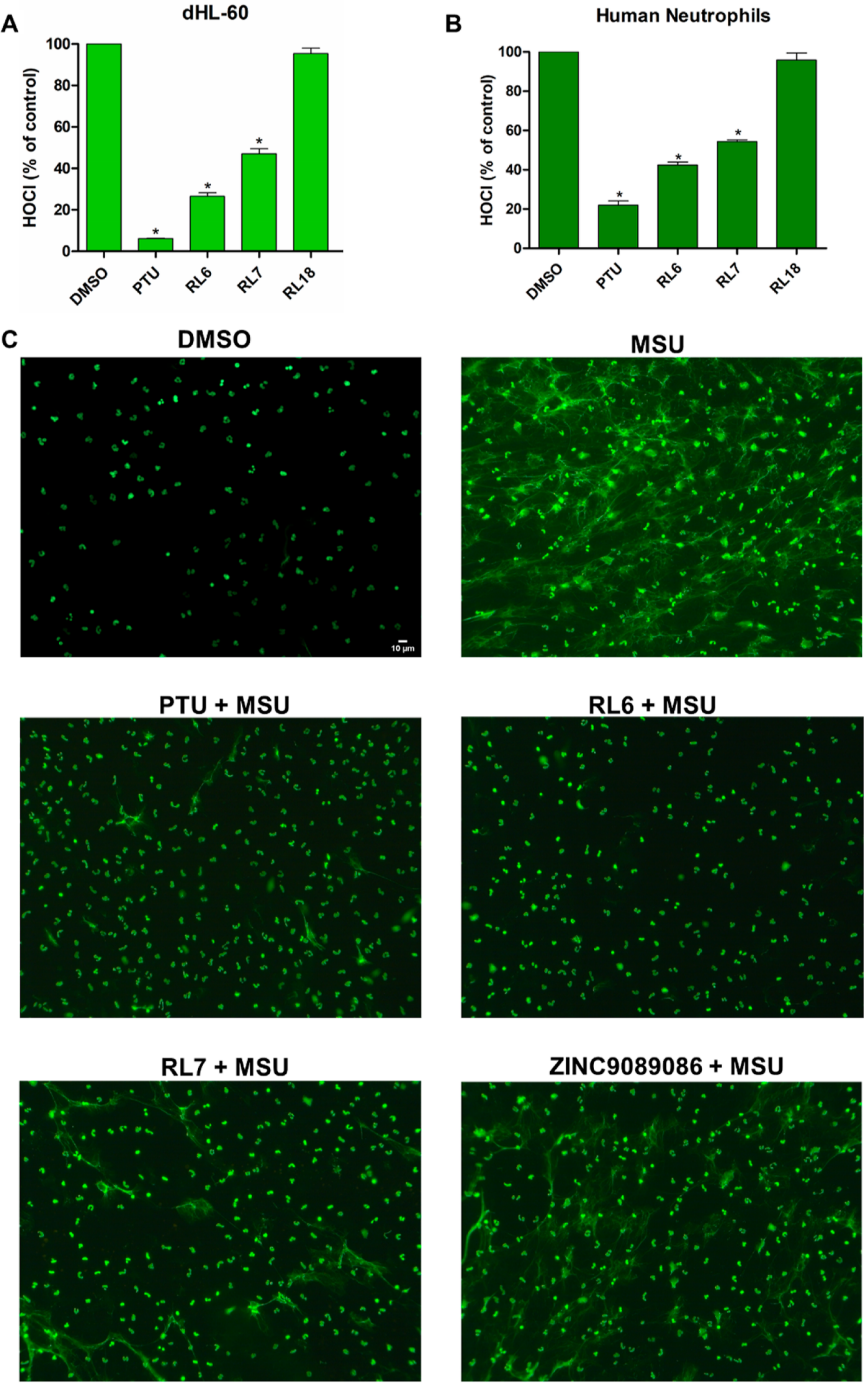
MPO inhibitors decrease HOCl and NETosis in cells. Inhibition of HOCl production by MPO inhibitors in the dHL-60 cell line (A) and human peripheral blood neutrophils (B). 1 ×10^6^ dHL-60 or neutrophils were incubated in PBS (10 mM Na_2_HPO_4_, 2 mM KH_2_PO_4_, 0.5 mM MgCl_2,_ 1 mM CaCl_2_, 140 mM NaCl, 5.5 mM dextrose), 100 μM DTPA, and 5 mM taurine in the presence of 20 μM compounds dissolved in 0.3% DMSO or 0.3% DMSO alone (control). Cells were activated with 100 nM PMA at 37 °C for 1 h. Then, the supernatant taurine chloramine was quantified by TMB. Inhibition of HOCl production was calculated as the percentage of control (vehicle, 0.3% DMSO). Statistical analyses were performed by one-way ANOVA, followed by Bonferroni’s posthoc test; *p < 0.01 from vehicle. Bars represent mean ± SEM of three independent experiments (*n* = 3). (C) Fluorescence microscopy of DNA stained with sytox green in nonstimulated (0.15% DMSO only) or urate monosodium crystal (MSU)-stimulated neutrophils. For the NETosis assay, adhered neutrophils were covered with RPMI medium containing 20 μM compounds and MSU (250 μg/mL). After 90 min of incubation, cells were fixed and kept at 4 °C overnight. Cells were then washed threefold with tris-HCl buffer (20 mM, pH 7.4), and DNA was stained by 500 μL of sytox green (500 nM) for 30 min. After being mounted and fixed on coverslips, the fluorescence images were acquired by the fluorescence microscope (λ_ex_ = 450–490 nm, λ_em_ = above 515 nm). Fluorescence microscopy is representative of at least three independent experiments. Additional frames of the independent replicates are presented in Figure S3A,B.

RL6 and RL7 significantly inhibited HOCl production in cultured neutrophil-like dHL-60 cells and peripheral blood neutrophils, while RL18 displayed no effect (Figure 6A,B). Since HOCl is produced in the extracellular milieu during PMA-induced oxidative burst, the inhibition is not related to cell membrane permeability. As expected, the irreversible inhibitor PTU was the most effective in counteracting HOCl production in both cell types (Figure 6A,B).

We investigated the effects of cell-active compounds, RL6 and RL7, along with the previously tested compound ZINC9089086,^31^ on the release of NETs. MSU crystals represent a classical inducer of aseptic NETosis,^48^ and MPO inhibitors are considered potential anti-NETotic agents.^49^ Our results showed that incubation of human neutrophils with MSU induced nuclear expansion and release of large DNA scaffolds (Figures 6C and S3A,B). The NETotic process was strongly inhibited by the MPO inhibitors PTU and RL6 and, to a lesser extent, by the HOCl scavenger RL7. While urate crystal-induced NETosis has been described as a reactive oxygen species-independent mechanism,^48^ our findings suggest that HOCl may contribute to this process.

### Off-Target Effect of RL6

RL6 is a derivate of 1,8-naphthalimide, and this chemical class is known for a range of biological activities.^50^ The 1,8-naphthalimide derivative, 4ANI, has been reported as a potent PARP1 inhibitor, with an IC_50_ value of 180 nM.^51^ PARP1 belongs to a family of proteins involved in DNA repair and genomic stability.^52^ While PARP1 inhibition may contribute to anti-inflammatory effects,^53^ many toxic effects are associated with the inhibition of this class of enzymes.^54^ Considering the high structural similarity between RL6 and 4ANI (Figure S4A), molecular docking simulations to compare their binding modes to the PARP1 active site were conducted. Following appropriate redocking, 4ANI makes hydrogen bonds with Gly863 and Glu988, as well as π stacking interactions with His862, Tyr896, and Tyr907 (Figure S4B, left). In contrast, RL6 presented a ∼ 45° rotation in the 1,8-naphtalimide plane, forming a nonanalogous hydrogen bond with Gly863, but maintained the same π stacking interactions (Figure S4B-right). Additionally, RL6 displayed two conformational clusters compared to one in 4ANI. However, RL6 exhibited a more favorable binding energy.

The differences in binding modes could be attributed to the presence of an additional amino group in the hydrazyl group and the distinct positioning of the amino group relative to the naphthalimide ring. These changes result in the exclusion of hydrogen bonds with Glu988 and the modification of the optimal interactions with Gly863. Furthermore, they discretely increased molecular dimensions and modified the molecular shape. To evaluate the inhibition of PARP1-induced ADP-ribosylation by RL6, RPE1-hTERT cells were incubated with H_2_O_2_. RL6 (1 μM, 10 μM and 50 μM) failed to inhibit H_2_O_2_-induced ADP-ribosylation. In contrast, the well-known PARP1 inhibitor, Olaparib,^55^ significantly decreased ADP-ribosylation (Figure S4C,D). This finding demonstrates that despite the structural similarities between 4ANI and RL6 molecules, RL6 does not act as an off-target inhibitor of PARP1. Although RL6 did not inhibit PARP1, it is crucial to consider this enzyme as a potential molecular target for MPO inhibitors selected by virtual screening.

### Toxicological Studies and Metabolism

Before conducting in vivo tests, compounds RL6, RL7, and ZINC9089086 underwent in silico and in vitro toxicological studies to evaluate their toxicological potential. Initially, acute toxicity was assessed using LD_50_ predicted by the Tox-Prediction server (https://comptox.charite.de/protox3/index.php?site=compound_input). The oral LD_50_ for RL6 was 1300 mg/kg, classifying it as risk 4 on a toxicity scale from 1 to 6 (6 indicating the absence of relevant toxicity). It is worth mentioning that this model utilizes structural similarity parameters, with RL6 being 61.04% similar to the compound with the experimental LD_50_ used for prediction. Additionally, the accuracy of this prediction was 68.07%. Compound RL7 exhibited a similar pattern of toxicity, with an LD_50_ value of 1000 mg/kg and a risk class of 4, albeit with lower similarity and accuracy parameters (42.74 and 54.26%, respectively). ZINC9089086 showed the lowest potential toxicity, with an LD_50_ value of 3000 mg/kg, classified as risk 5, and presenting similarity and accuracy parameters of 54.13 and 67.38%, respectively. It is worth highlighting that all three compounds underwent predictive models for metabolization by enzymes CYP1A2, CYP2C19, CYP2C9, CYP2D6, CYP3A4, and CYP2E1 that are included in the Tox-Prediction server. Only RL7 showed a slight probability of being a CYP2D6 substrate (0.54%).

Additionally, RL6 displayed no cytotoxicity to hTERT-RPE1 cells, whereas RL7 was highly cytotoxic at 50 μM (Figure S5).

### Inhibition of Paw Edema in a MSU-Induced Experimental Gouty Arthritis in Mice

**Figure 7:**
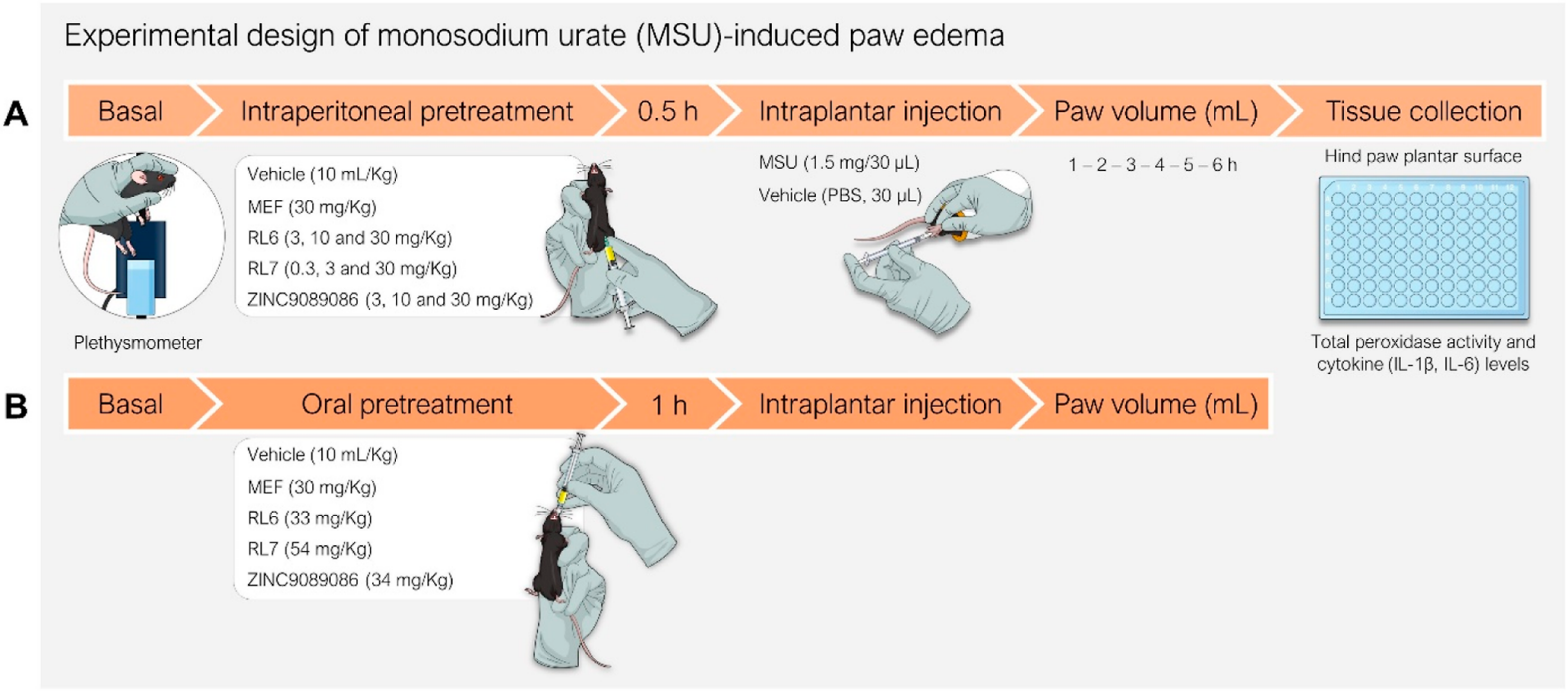
Flowchart of the MSU-induced paw edema experiment. Design for intraperitoneal (A) or oral (B) treatment.

**Figure 8:**
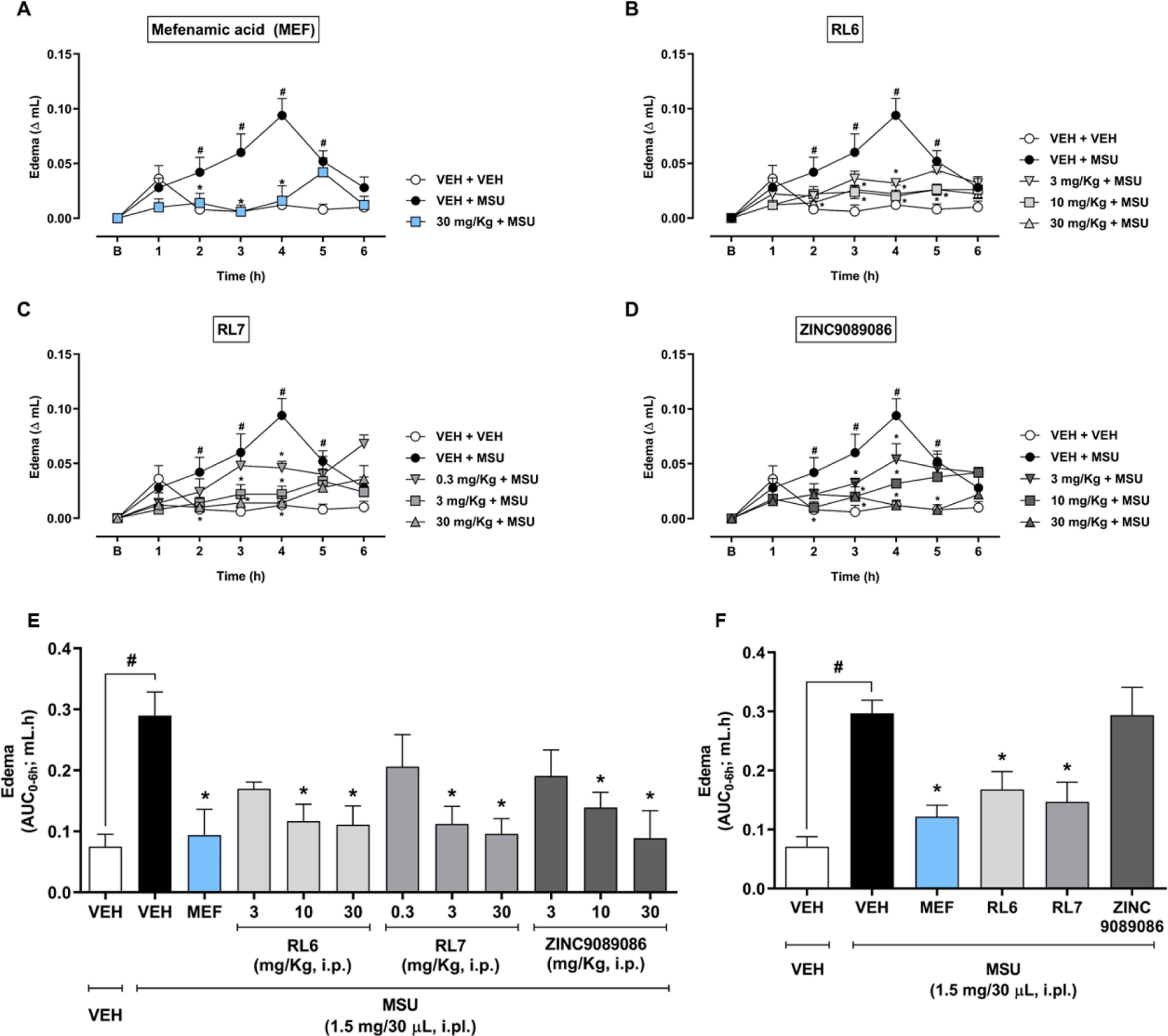
Antiedematogenic effect of MPO inhibitors and HOCl scavenger in MSU-induced paw edema. Mice were pretreated with vehicle (VEH: 5% DMSO; 0.5% Tween-80 in PBS, i.p.), mefenamic acid (A, MEF: 30 mg/kg, i.p.), RL6 (B, 3, 10, and 30 mg/kg, i.p.), RL7 (C, 0.3, 3, and 30 mg/kg, i.p.), or ZINC9089086 (D: 3, 10, and 30 mg/kg, i.p.), and the respective AUCs are shown in E. (F) Antiedematogenic effect of the oral administration of MPO inhibitors and HOCl scavengers in MSU-induced paw edema, RL6 (33 mg/kg, po), RL7 (54 mg/kg, po), ZINC9089086 (34 mg/kg, po), and MEF (30 mg/kg, i.p.). After 30 min, mice received vehicle (VEH: PBS, 30 μL, i.pl.) or MSU (1.5 mg/30 μL, i.pl.). Paw volume (mL) was evaluated before (B, baseline) and after pretreatments for 6 h in a plethysmometer. Statistical analyses were performed by two-way ANOVA, followed by Bonferroni’s posthoc test; ^#^*P* < 0.05 compared to the “VEH + VEH” group; *P < 0.05 compared to the “VEH + MSU” group, *n* = 5/group.

The potential anti-inflammatory effects of RL6, RL7, and ZINC9089086 were investigated in an in vivo murine model of gouty arthritis (Figure 7).^22^ Intraplantar injection of MSU led to an increase in paw volume (VEH + MSU: 0.29 ± 0.03 mL), when compared to the animals that received intraplantar PBS (VEH + VEH: 0.07 ± 0.02 mL; Figure 8) The time-dependent edema exhibited an inverted V-shaped curve, similar to the previously reported study.^22^ However, in our experiments, the maximum edematogenic response occurred at the fourth hour instead of the sixth. Intraperitoneal treatment with the nonsteroidal anti-inflammatory drug (NSAID), mefenamic acid (30 mg/kg), prevented paw edema for up to 4 h (Figure 8A). RL6, at doses of 3, 10, and 30 mg/kg, significantly prevented paw edema, with a long-lasting effect observed at the two higher doses used (Figure 8B).

The HOCl scavenger RL7, and the previously described MPO inhibitor ZINC9089086, also inhibited paw edema at all of the tested doses. At the lowest dose (0.3 mg/kg), RL7 had a significant effect only at the peak of paw edema (4th hour), but it was effective from 1 to 4 h at doses of 3 and 30 mg/kg (Figure 8C). ZINC9089086, at all tested doses, inhibited paw edema up to 4 h, with a long-lasting effect at the highest dose (Figure 8D).

Time-dependent area under the curve revealed that mefenamic acid (30 mg/kg) reduced paw edema by 91.1% when compared to the “VEH + MSU” group. RL6 significantly decreased the paw volume by 80.4 and 83.2% at doses of 10 and 30 mg/kg, respectively, while RL7 reduced it by 82.8 and 90.2% at doses of 3 and 30 mg/kg, respectively. ZINC9089086 displayed a significant effect only at the highest dose of 30 mg/kg, reducing paw edema by 93.4% (Figure 8F).

We then selected the highest dose of the compounds to test their inhibitory effect when administered by an oral route. For a meaningful comparison, the doses of the compounds were adjusted to an equimolar concentration. As expected, intraplantar administration of MSU increased the paw volume (VEH + MSU: 0.29 ± 0.02 mL) compared to the “VEH + VEH” group (0.07 ± 0.01 mL). RL6, RL7, and the positive control, mefenamic acid, inhibited edema by 57.0, 74.3, and 77.3%, respectively, compared to the “VEH + MSU” group (Figure 8G). However, ZINC9089086 showed no significant inhibition. This result confirmed that, by filtering compounds using Lipinski–Veber parameters,^31^ molecules with suitable oral bioavailability were truly recovered.

### RL6 Decreases Tissue Peroxidase Activity and IL-1β Levels

**Figure 9:**
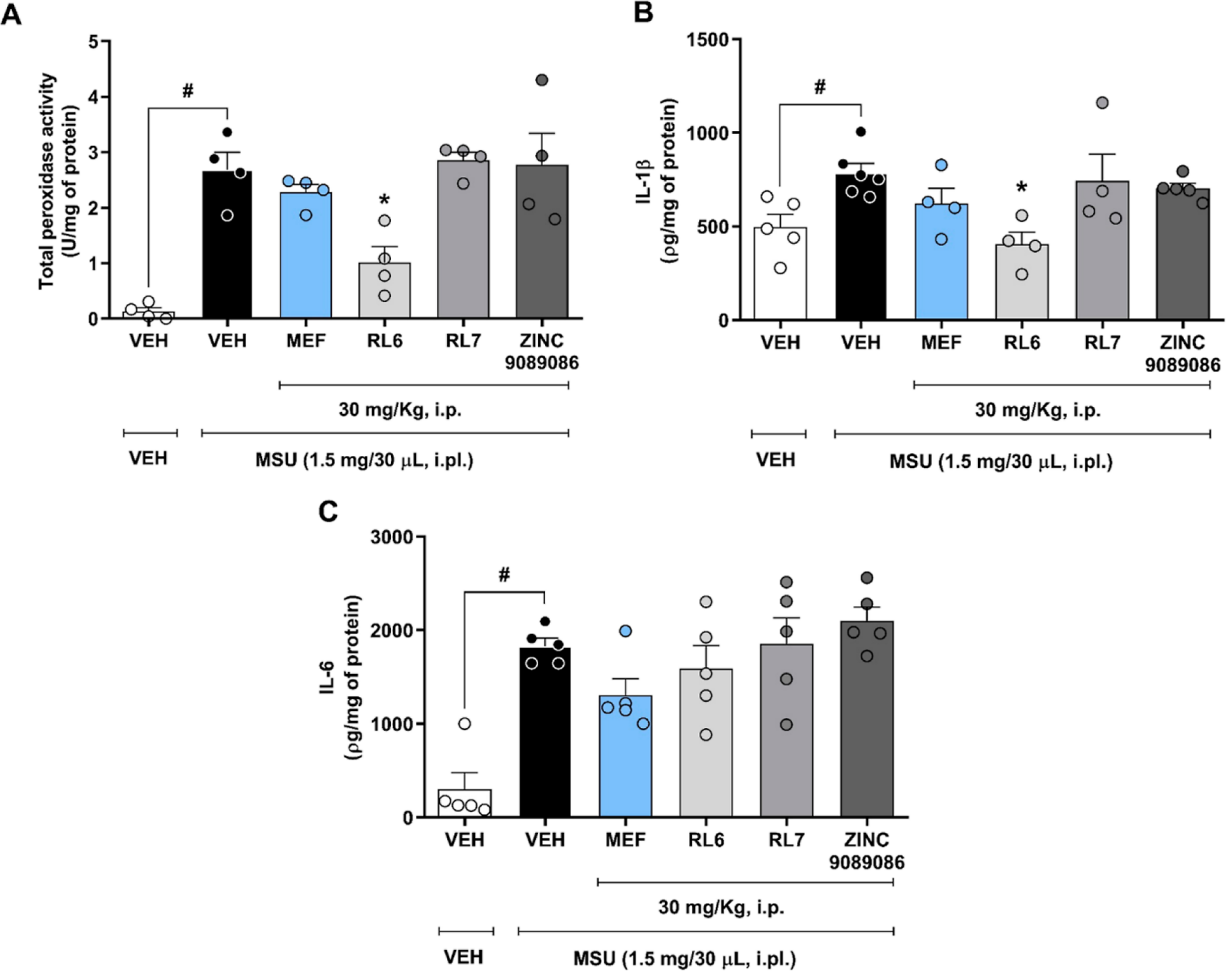
Effect of intraperitoneal treatment with MPO inhibitors and the HOCl scavenger on the total peroxidase activity (A), and IL-1β (B) and IL-6 (C) levels in the hind paw tissue. Mice were treated as described in Figure 6. After the last paw volume measurement (6 h), the hind paws were collected and tissue were prepared for total peroxidase activity and cytokine assessment. Statistical analyses were performed by one-way ANOVA, followed by Bonferroni’s posthoc test; ^#^*P* < 0.05 compared to the “VEH + VEH” group; *P < 0.05 compared to the “VEH + MSU” group, *n* = 4–5/group.

Considering that MSU-induced arthritis increases total peroxidase and IL-1β and IL-6 levels,^22^ these inflammatory markers were quantified at the end point of paw edema measurement. All three markers were found to be significantly increased in the MSU group (Figure 9). Intraperitoneal pretreatment with RL6 (30 mg/kg) led to a substantial reduction of 61.5% in the total peroxidase activity and a 48.3% decrease in IL-1β levels, while it had no significant impact on IL-6 levels. In contrast, RL7, ZINC9089086, and mefenamic acid were unable to decrease these proinflammatory parameters, possibly due to their short-lasting effects.

## Discussion

This paper presents four key findings: first, integrating the MPO inhibitor-like rule with a validated structure-based virtual screening method^31^ offers an effective strategy to identify putative and chemically diverse MPO inhibitors. Second, our mechanistic studies unveiled distinct mechanisms for the two most potent hits discovered. Third, the identified MPO inhibitors also decreased the production of HOCl by stimulated dHL-60 and neutrophils as well as MSU-induced NETosis. Fourth, pretreatment with the compounds, whether administered intraperitoneally or orally, effectively prevented paw edema in a murine model of MSU-induced gouty arthritis.

Initially, we carried out a revalidation of the structure-based virtual screening approach, which involved aligning new MPO crystal structures to confirm the limited flexibility of residues with the active site (Figure S1).^31^ While previous studies also utilized this rigid receptor configuration,^28−30^ our approach differed by employing heme atomic charges calculated using the PM7 Hamiltonian. This novel approach enhanced the ability to recover ligands in experimental conformations during cross-docking validation simulations, thereby improving the efficacy of the structure-based virtual screening step. The use of PM7 to describe the electronic properties of the MPO active site, including the high-spin Fe(III) and a sulfonium ion, demonstrated the utility of this semiempirical quantum method in facilitating the development of new virtual screening methodologies reliant on atomic charges. The robustness of this methodology was further confirmed by the fact that a majority of compounds inhibited both chlorinating and peroxidatic activities, yielding one of the highest hit rates reported in the search for MPO inhibitors (65 and 70%, respectively).^28−30^ It is crucial to assess both MPO activities to validate the inhibitory activity of the computational hits, as exclusive peroxidase inhibition may hold less physiological relevance given the abundance of chloride ions.^23,52,53^ Conversely, exclusive chlorinating activity inhibition suggests that a compound may function as a scavenger of HOCl rather than a true enzyme inhibitor. Of note, the MPO inhibitor-like rule demonstrated its ability to facilitate bioisosteric approaches. For instance, RL6 closely resembles the known inhibitor ABAH and the antituberculosis drug isoniazide.^36,40,56^

Molecular docking analyses indicate that the most prevalent interaction at the MPO active site involves π stacking interactions with the heme group. This is closely followed by the formation of hydrogen bonds with Arg233, heme carboxylate, His95, Gln91, and Glu102. It is noteworthy that the occurrence of a hydrogen bond with Arg233, previously unreported as a frequent interaction with MPO inhibitors,^28,30,31,57^ implies that our methodology identifies inhibitors with distinct binding modes. Additionally, molecular docking simulations showed that four inhibitors, RL9, RL20, RL26, and RL27, exhibit a sulfur atom in close proximity to the heme carboxylate, adjacent to Glu102 and Thr100 residues. This finding suggests that this specific region of the active site possesses stereochemical complementarity for sulfur. This information confers potential insights into the development of more potent and selective MPO inhibitors. The formation of π stacking interactions and multiple hydrogens bonds, including an orientation near to His95 that mirrors the transition state observed in the hydrogen peroxide–MPO complex,^37^ can account for the nanomolar potency observed for RL6. This compound, a 1,8-naphthalimide derivative, belongs to a chemical class known by diverse biological activities, including anticancer, antibacterial, antiviral, and antiprotozoal.^50^ Furthermore, the higher aromaticity for RL6 compared to that of ABAH, as indicated by the aromaticity index HOMA, supports its nanomolar potency. This property facilitates the binding and persistence of the compound within the MPO active site. Therefore, the inclusion of aromaticity studies may enhance the profile of MPO-like inhibitors in future research endeavors.

When the significantly higher ratio of chlorinating to peroxidase activity was compared for RL7, it would be expected that RL7, unlike RL6, could act as a HOCl scavenger. This hypothesis was confirmed by the rapid fluorescence decrease of RL7 in the presence of HOCl. HOCl can react with amino groups to form chloramines,^58^ as well as with thiophene sulfur, which can be oxidized by multiple HOCl equivalents.^59^ RL6 presented a chlorinating/peroxidase activity ratio of 1, suggesting its role as an enzyme inhibitor. The absence of RL6 oxidation by MPO/H_2_O_2_ or MPO/H_2_O_2_/Cl^–^ suggests that RL6 neither acts as a scavenger of HOCl nor serves as a substrate for MPO. While most known MPO inhibitors are reversible inhibitors that function as substrates for compound I, thereby trapping the enzyme as compound II, or are irreversible suicidal inhibitors that become attached to the enzyme after oxidation by compound I,^60^ RL6 does not fit into either of these categories. Importantly, RL6 is a reversible inhibitor (Figure 3C), but it does not act as a substrate for compound II. In concordance with our hypothesis, inhibitory activity is not lost even in the presence of tyrosine, a substrate for compound II. Due to RL6’s high absorption at 413 nm, it remains uncertain whether RL6 binds tightly to the native enzyme or to the compound I intermediate. The reversible and nonoxidizable mechanism exhibited by RL6 was unexpected given its structural analogy to the classic irreversible inhibitor ABAH,^36,40^ suggesting a behavior more akin to recently discovered triazolopyridines.^27^ Thus, future kinetic studies are necessary to determine the exact mechanism of inhibition.

Although cell-free assays can be employed as a first simpler way to validate computational hits, cellular and functional assays are necessary to take into account membrane permeability, hydrophobicity, and binding to unspecific targets.^61,62^ Additionally, in the cellular milieu, inhibitors compete with different enzyme substrates. For instance, neutrophil oxidative burst simultaneously produces both MPO substrates, superoxide and hydrogen peroxide, which lead to the formation of different MPO intermediates.^14,63−65^ RL6 and RL7 were still able to inhibit HOCl in cells, overcoming the presence of other substrates. In contrast, the inhibitory effect of RL18 was lost when tested in the cells.

Despite the fact that mechanisms of NET release and content can vary depending on the stimulus, active MPO seems to be important for NETosis in most cases,^16−18,66^ including in response to MSU.^48^ In support of that, RL6 and the irreversible MPO inhibitor, PTU, decreased NET formation induced by MSU. Importantly, the MPO reversible inhibitor ABAH failed to inhibit MSU-induced NETs, possibly due to the partial inhibition of the enzyme.^48^ Scavenging of HOCl by RL7 was not enough to contain NET formation and release, suggesting that an active MPO rather than its final product HOCl is important for the NETosis process.

Conflicting results have emerged regarding the role of MSU-induced NETosis in resolving gout inflammation. Some studies suggest that NETs, which contain proteases, degrade inflammatory cytokines and play a central role in resolution.^21^ However, these results, obtained from isolated human neutrophils, were not replicated in a murine model of MSU-induced gout in vivo.^22^ One should consider that in vitro assays, using isolated neutrophils, may not fully represent the complex tissue interactions involving macrophages in the resolution process. Interestingly, we show that both genuine MPO inhibition (RL6) and HOCL scavenging (RL7) can prevent acute paw edema, despite their different ability to counteract NET formation. These results suggest that MPO inhibition and HOCl scavenging can confer tissue protection by independent mechanisms.

It is noteworthy that at higher doses (10 and 30 mg/kg) the three tested compounds presented a similar effect to 30 mg/kg mefenamic acid, an NSAID used to treat pain and inflammation in gout. However, NSAIDs have been widely used in clinical practice for the treatment of gouty arthritis.^67^ Prolonged use of mefenamic acid and other cyclooxygenase-2 (COX-2) inhibitors are often associated with gastrointestinal (ulcers, intestinal villous atrophy, diarrhea, and bleeding) and cardiovascular issues.^68^ Because of this, the search for a safer and more effective therapeutic alternative is still necessary. Of relevance, RL6 and RL7 presented in vivo efficacy even though no structural optimizations were carried out, and such effect is uncommon for initial, nonoptimized hits.^69^ Additionally, both RL6 and RL7 demonstrated oral bioavailability, indicating their potential for oral administration. These compounds were screened using the MPO-inhibitor like rule, which satisfies classic Lipinski rules for oral absorption and bioavailability. Two out of three compounds, RL6 and RL7, met these criteria, validating the MPO inhibitor-like rule as an effective approach for discovering orally active compounds.^31^ RL6 was the sole intervention that demonstrated a notable reduction in both total peroxidase levels and IL-1β expression within the paw tissues, being superior even to the NSAID mefenamic acid. Furthermore, RL6 exhibited a modest, although statistically nonsignificant, reduction in IL-6 production and release. The release of IL-1β is a well-established response to inflammasome activation by urate crystals.^2,70,71^ Thus, modulation of IL-1B levels by an MPO inhibitor would bring therapeutic benefits, considering the role of this cytokine in the pathogenesis of gouty arthritis. In contrast, the release of IL-6 has received relatively less attention. However, it is worth noting that elevated levels of IL-6 have been observed in mouse serum during the induction of MSU-induced gouty arthritis.^72^ Moreover, in the context of human studies, there is a correlation between serum IL-6 levels and the presence of gout tophi and deformities, as previously reported.^73^

Despite the structural similarity between RL6 and the PARP1 inhibitor, 4ANI, and RL6’s superior binding energy to the PARP1 active site, RL6 proved ineffective in counteracting ADP ribosylation. This finding conclusively demonstrates that RL6’s anti-inflammatory effects are not linked to PARP1 inhibition.^53^ While this approach provided valuable insights into specificity, we cannot neglect a possible effect upon other inflammatory signaling pathways. Considering RL6’s impressive in vivo effects and its low molecular weight, studies on structure–activity relationships are encouraged, aiming to further increase its potency concomitantly with its solubility and to decrease planarity, as this molecule may intercalate to DNA, generating undesirable toxic effects by chronic use. Although no specific toxicological studies were carried out, no acute toxic effects were observed during all tests.

## Conclusions

In summary, RL6 stood out as the most promising MPO inhibitor for the next steps in drug development due to several key attributes. First, it acts as a reversible MPO inhibitor. Reversible inhibitors typically exhibit a lower toxicity. Second, it is not a compound II accumulator, ensuring effectiveness even in the presence of endogenous physiological substrates for compound II. Third, it inhibits HOCl generation by neutrophils and reduces NETosis, paw edema, total peroxidase activity, and IL-1β release in a murine model of gout arthritis. Fourth, RL6 boasts good oral availability and has shown no sign of acute toxicity. Altogether, these results underscore the effectiveness of integrating an inhibitor-like rule and structure-based virtual screening as a gold methodology for discovering orally active MPO inhibitors with anti-inflammatory effects in a gout model of arthritis. The application of this methodology to new databases, such as ZINC20,^74^ promises a more comprehensive exploration of chemical space and offers a pathway to uncover novel chemotypes targeting MPO. This, in turn, enhances and diversifies the discovery of new preclinical candidates with potential therapeutic values.

## Experimental Section

### Materials

Human leukocyte MPO (EC 1.7.1.11) was purchased from Planta Natural Products (Vienna, Austria). Inhibitors were purchased from Specs (Zoetermeer, The Netherlands). All compounds presented purity between 90 and 95% determined by LC/UV–vis, LC/MS, or NMR (Figure S6). Inhibitor stock solutions (12 mM) were prepared in dimethyl sulfoxide (DMSO), stored at −20 °C, and diluted in phosphate buffer (10 mM, pH 7.4) at the time of use. Hydrogen peroxide working solution (Merck/Sigma, Germany) was prepared at the time of use, and the concentration was calculated by measuring the absorbance at 240 nm (ε_240_ nm = 43.6 M^–1^ cm^–1^).^75^ Phosphate buffer (10 mM, pH 7.4) was used in the presence of 100 μM DTPA (diethylenediamine pentaacetic acid). Cytokines were assessed by ELISA (BioLegend, CA, USA) and protein content using Bradford (1976) (Bio-Rad, Hercules, CA, USA).^76^ SYTOX and ProLong Diamond Antifade Mountant were purchased from Invitrogen (Massachusetts, USA). Cell Proliferation Kit II (XTT) was purchased from ROCHE (code: 11465015001). All other reagents were from Sigma-Aldrich (Darmstadt, Germany) unless stated otherwise.

### Virtual Screening

A virtual sublibrary composed by 242 potential MPO inhibitors was used for the selection of compounds. This set was obtained from our previous studies using the Zinc12 database containing more than 35 million compounds. The library was filtered by an inhibitor-like rule and docked into MPO active site using the GOLD version 5.4 molecular docking program.^31^ The molecular docking methodology was re-evaluated through the alignment of new reported MPO structures (PDB 5QJ2, 5QJ3, 6WXZ, 6WY0, 6WY5, 6WY7, 6WYD, 7LAE, 7LAG, 7LAL, and 7LAN) using Pymol 1.8 and Chimera 1.10.1 visualization programs. The alignment of MPO structures was used to re-evaluate the presence of conserved water molecules and the flexibility of active site residues by visual inspection. The protonation and conformation of active site residues in PDB 1CXP were adjusted as well as the atomic charges of the heme group, which were calculated by the PM7 Hamiltonian as recently published.^31^ Molecular docking simulations with AutoDock 4.2.3 were re-evaluated by cross-docking using PDB 7LAG and 7LAN ligands and docked into the PDB 1CXP MPO active site, as previously described.^31^ The refRMS values between the poses and the experimental conformations were calculated by AutoDock 4.2.3. The computational hits were selected using the following criteria: hydrogen bonds number, presence of π stacking interactions, histogram profile, and binding energy.^31^ The lower energy conformation inside the most populous cluster was used to select the bioactive pose to be analyzed.

### Chlorinating Activity Assay

Chlorinating activity was carried out in phosphate buffer (20 mM, pH 7.4). Compounds (20 μM) were dissolved in 0.03% DMSO, MPO (10 nM), diethylenetriaminepentaacetic acid (100 μM), cetyltrimethylammonium chloride (0.03%), NaCl (140 mM), and taurine (5 mM).^31^ This mixture was incubated at 37 °C for 15 min before adding 40 μM hydrogen peroxide. The reaction ran for 8 min and was then stopped by catalase (20 μg/mL). Oxidation of TMB (2 mM) (3,3,5,5-tetramethylbenzidine) by taurine chloroamine catalyzed by iodide (100 μM) was quantified at 650 nm.^33^ Results were expressed as a percentage of the control (vehicle). IC_50_ was calculated by nonlinear regression using the Hill equation present in GraphPad Prism 5 software.

### Peroxidatic Activity Assay

MPO peroxidase activity was kinetically monitored using the artificial peroxidase substrate AmplexRed, which is oxidized to resorufin and can be detected by fluorescence.^34^ Reactions were carried out in phosphate buffer (50 mM, pH 7.4), MPO (100 pM), cetyltrimethylammonium chloride (0.03%), diethylenetriaminepentaacetic acid (100 μM), AmplexRed (30 μM), and compounds (20 μM in 0.3% DMSO). The reaction was started by adding 2 μM hydrogen peroxide. The reaction product was assessed by fluorescence at λ_ex_ = 530 and λ_em_ = 580 nm. The initial velocity was calculated by the slope of the linear regression in the first minutes of reaction. Inhibition is represented as a percentage of the control (vehicle).

### Taurine Chloroamine Scavenger Assay

Taurine chloramine was prepared by mixing hypochlorous acid (2.81 mM) with taurine (5 mM), cetyltrimethylammonium chloride (0.03%), NaCl (140 mM), and diethylenetriaminepentaacetic acid (100 μM) in phosphate buffer (20 mM, pH 7.4). The system was kept at 37 °C for 5 min for complete chloramine taurine formation. The compounds were added to this mixture and incubated for 15 min at 37 °C. Taurine chloramine was quantified by the oxidation of TMB (2 mM) catalyzed by iodide (100 μM).^33^

### Reversibility Test

Reversibility of the inhibition was analyzed by peroxidase residual activity.^77^ MPO (100 nM) was incubated with inhibitors (20 μM, 0.33% DMSO) in phosphate buffer (20 mM, pH 7.4) containing cetyltrimethylammonium chloride (0.03%) and hydrogen peroxide (40 μM) at 37 °C for 30 min. After incubation, the system was diluted 200-fold using acetate buffer (200 μM, pH 5.4) in the presence of TMB (2 mM dissolved in 10% dimethylformamide). After a new hydrogen peroxide addition (2 μM), the oxidation of TMB was quantified at 655 nm.

### Kinetics for the Oxidation of the Compounds

Changes in the absorption and fluorescence spectra of RL6 and RL7 were determined by incubating 1 μM compounds in 0.3% DMSO and phosphate buffer (20 mM, pH 7.4) with or without MPO (10 nM), diethylenetriaminepentaacetic acid (100 μM), taurine (5 mM), cetyltrimethylammonium chloride (0.03%), and NaCl (140 mM). In the chloride-free system, NaCl and taurine were omitted, and cetyltrimethylammonium chloride was replaced by cetyltrimethylammonium bisulfate. The reaction was performed in the absence or presence of Tyr (50 μM) and was started by adding 40 μM hydrogen peroxide. RL6 and RL7 consumption was monitored by fluorescence at λ_ex_ = 413/λ_em_ = 603 nm for RL6 or λ_ex_ = 306/λ_em_ = 603 nm for RL7 in a microplate reader.

The fast oxidation of RL7 was monitored in a stopped-flow spectrophotometer (Applied Photophysics SX20) using the intrinsic RL7 fluorescence (λ_ex_ = 306 nm and λ_em_ = above 340 nm). The reaction of RL7 (20 μM in 0.3% DMSO) with HOCl (250 μM) was performed in phosphate buffer (20 mM, pH 7.4), diethylenetriaminepentaacetic acid (100 μM), and NaCl (140 mM). Data were obtained within 1 s.

### MPO Spectra

MPO (0.5 μM) spectra were monitored in phosphate buffer (10 mM, pH 7.4) in the presence or absence of 10 μM RL6 and 50 μM tyrosine. Reactions were started by adding 100 μM H_2_O_2_. Spectra were taken in a Cary 60 UV–vis spectrophotometer (Agilent, CA, EUA).

### HOMA Calculation

The geometries of RL6 and ABAH were optimized by the B3LYP/6-31G(d) method. The aromatic index HOMA (harmonic oscillator model of aromaticity) was calculated using Multiwfn 3.8 software.^78^

### Cell Culture

Human promyelocytic leukemic cells (HL-60) (BCRJ, RJ, Brazil) were cultivated in RPMI with fetal bovine serum (10%), penicillin (62 μg/mL), and streptomycin (100 μg/mL) at 37 °C in 5% CO_2_ atmosphere. HL-60 cells were differentiated to neutrophil-like (dHL-60) by DMSO (1.25%) during 4 days.^25^

### Peripheral Blood Neutrophils

Human neutrophils were obtained from healthy individuals as previously described.^24^ The experiments were approved by the local ethics committee (CEPSH-ICB 1435/18).

### Preparation of Urate Crystals

In sterile conditions, 1 g of crystalline uric acid was mixed with 200 mL of sterile ultrapure water. The mixture was solubilized by shaking and 20% NaOH addition until complete solubilization.^79^ In the next step, the solution was heated to 70 °C, and pH was adjusted to 7.4 by adding HCl. Finally, the solution was slowly cooled. Crystals were vacuum-dehydrated overnight and kept in a desiccator containing anhydrous silica gel until use. Crystals were resuspended in sterile PBS immediately before use.

### Cellular Production of HOCl

Before any assay, the oxidative burst of dHL-60 or neutrophils was evaluated by incubating the cells with phorbol myristate acetate (100 nM). The reduction of cytochrome *c* due to the production of superoxide was monitored over time at 550 nm and ε = 21,000 M^–1^ cm^–1^.

Measurement of HOCl production by 1 × 10^6^ dHL-60 or neutrophils was carried out in PBS (10 mM Na_2_HPO_4_, 2 mM KH_2_PO_4_, 0.5 mM MgCl_2,_ 1 mM CaCl_2_, 140 mM NaCl, 5.5 mM dextrose, 100 μM diethylenetriaminepentaacetic acid, 5 mM taurine) in the presence of the compounds (20 μM in 0.3% DMSO) or 0.3% DMSO (control). Cells were activated with phorbol myristate acetate (100 nM) at 37 °C for 1 h. After incubation, cells were centrifuged at 1400 rpm for 10 min, and the supernatant was diluted threefold. Finally, 240 μL of this supernatant was added to 60 μL acetate buffer (400 mM, pH 5.4) containing NaI (100 μM) and TMB (2 mM in DMFO 10%). Taurine chloramine was quantified by TMB oxidation at 655 nm.^33^ Inhibition of HOCl production was calculated as the percentage of the control (vehicle).

### NETosis Experiment

NETosis assay was conducted on human neutrophils adhered to lysine-coated glass coverslips as previously reported.^80^ After neutrophils’ adhesion, the compounds were diluted in RPMI medium (free of phenol red and fetal bovine serum) at a final concentration of 20 μM (0.15% DMSO) and added to the cells for 15 min at 37 °C in a 5% CO_2_ atmosphere. Then, MSU crystals (250 μg/mL), known to induce NETosis, were added. After 90 min incubation, the medium was carefully removed, paraformaldehyde was added (500 μL at 4%), and incubated at 4 °C overnight. In the following day, paraformaldehyde was removed, and cells were washed threefold with tris-HCl buffer (20 mM, pH 7.4) and kept into the same buffer at 4 °C. Tris-HCl buffer was carefully removed, and DNA was stained by adding 500 μL SYTOX Green (500 nM diluted in tris-HCl buffer). The system was kept for 30 min at room temperature, protected from light. After staining, the coverslips were mounted over the glass slides using ProLong Diamond Antifade Mountant fixation medium. Fluorescence images were acquired in an Axiovert 200 (Zeiss) fluorescence microscope with a 20*x*/0.4 objective and Zeiss Filter Set 09, λ_ex_ = 450–490 nm, λ_em_ = above 515 nm. Images were taken by an Axiocam HR R3 camera device.

### PARP1 Off-Target Assay

RPE1-hTERT cells were grown at 37 °C in a humidified atmosphere containing 5% CO_2_ in DMEM-F12 medium supplemented with 10% fetal bovine serum and penicillin/streptomycin at 100 U/mL and 100 μg/mL, respectively (15140122, Thermo). Cells (10^5^ per well) were seeded in a 12-well plate, and 24 h later, fresh medium containing 10 μM PARGi (PDD00017273-Sigma) and DMSO, 10 μM olaparib (Selleckchem), or 1, 10, or 50 μM RL6 was added. After 1 h of pretreatment, the cells were treated with 600 μM H_2_O_2_ (Sigma) in PBS for 10 min at 37 °C. Cells were lysed in preheated Laemmli buffer devoid of beta-mercaptoethanol and bromophenol blue, and the lysates were boiled for 15 min. Protein concentration was determined using a BCA protein quantification kit (Pierce) and normalized. Beta-mercaptoethanol and bromophenol blue were then added, and the lysates were boiled again for 15 min. Proteins were separated in a 10% SDS-PAGE gel and then transferred to nitrocellulose membranes (Bio-Rad). After staining with Ponceau Red and trimming, membranes were blocked with 5% milk for 1 h and incubated with the pan-ADP-ribose binding reagent (1:1000, MABE1016, Millipore) at 4 °C overnight, followed by incubation with secondary antirabbit-HRP antibody (1:5000, SAB3700934-2MG, Sigma) for 1 h. Membranes were incubated with ECL Prime (Amersham), and the signals were detected using a Chemidoc MP Imaging System (Bio-Rad). Membranes were then reprobed with antiactin primary antibody (1:5000, MAB1501, Sigma) and secondary antimouse-HRP antibody (1:5000, SAB3701105-2MG, Sigma), followed by ECL incubation and imaging as described above. Signals were quantified using ImageJ software.

### Cell Viability Assay

hTERT-RPE1 cells were seeded in 24-well plates at 2 × 10^4^ cells per well. After 24 h, cells were treated with 2, 5, 10, 25, or 50 μM RL6, RL7, or PTU in a culture medium (DMEM/F-12 with HEPES and 10% FBS). DMSO was added to untreated cells at 0.42% concentration as a vehicle control. After 72 h of treatment, cells were washed with PBS and left in 200 μL of XTT working solution for 3 h. The working solution was prepared according to manufacturer’s instructions but diluted eightfold in PBS. The supernatant was transferred to a 96-well plate; absorbance was read at 450 and 750 nm in a Synergy (Biotek, Winooski, VE, USA) plate reader and presented as percentage of control.

### Animals and Experimental Design

All experiments were carried out using male C57BL/6 mice (7–8 weeks old, 20–25 g). These mice were provided from the local vivarium at the Faculty of Medicine, University of São Paulo, Brazil. The mice were housed in polypropylene cages, with a maximum of five mice per cage. They were maintained under controlled conditions, including controlled temperature (22 ± 2 °C) and luminosity (12 h light/dark cycle), air exhaustion, and free access to water and food (Nuvilab CR-1, Quimtia S/A, Brazil). Prior to conducting the experiments, all experimental protocols were reviewed and approved by the local Ethics Committee of Animal Experimentation (CEUA/IQ, n° 100/2018). The experiments were strictly carried out in agreement with the guidelines set by the Brazilian Council for Control of Animal Experimentation (CONCEA) and the principles of the National Council for Animal Experimentation Control, consistent with the Animal Welfare Act.

### MSU-Induced Paw Edema

MSU-induced edema was performed as previously described.^22^ The compounds were prepared by dilution in a solution containing 0.5% Tween 80 and 5% DMSO and then resuspended in PBS prior to administration. The animals were treated intraperitoneally (i.p.) with the following substances: vehicle (VEH: 5% DMSO; 0.5% Tween 80 in PBS), mefenamic acid (a nonsteroidal anti-inflammatory drug at a dosage of 30 mg/kg), RL6 (at doses of 3, 10, and 30 mg/kg), RL7 (at doses of 0.3, 3, and 30 mg/kg), or ZINC9089086 (at doses of 3, 10, and 30 mg/kg).^31^ After 30 min, the mice were anesthetized using inhaled isoflurane (2% v/v in O_2_). A hypodermic syringe with a 31-gauge needle (BD, Franklin Lakes, NJ, USA) was used to administer the vehicle (VEH: PBS, 30 μL) or MSU (1.5 mg/30 μL) via the intraplantar route (i.pl.), into the right hind paw (Figure 9).

Paw volume (mL) was measured at various time points, including before injection (baseline, B), 1, 2, 3, 4, 5, and 6 h ,and at 1, 2, 3, 4, 5, and 6 h following MSU injection using a plethysmometer (Ugo Basile, Comerio, Italy) (Figure 7). For this, the injected hind paw was immersed into the dipping solution (6 mM NaCl diluted in distilled water and added with 2 mL/L of a wetting compound provided by the manufacturer) until the ankle. The extent of edema was quantified as a change in paw volume (Δ mL) by subtracting the basal paw volume from the volume of the paw measured at each time point. The extent of edema was quantified as a change in paw volume (Δ mL). To evaluate the antiedematogenic effect of orally administered MPO inhibitors (p.o.), the animals were fasted for 1 h and then treated with equimolar doses of the following substances: vehicle (VEH: 5% DMSO; 0.5% tween 80 in PBS), mefenamic acid (30 mg/kg), RL 6 (33 mg/kg), RL 7 (54 mg/kg), RL 21 (30 mg/kg), or ZINC9089086 (34 mg/kg). After 1 h, the anesthetized mice (2% isoflurane, v/v in O_2_) received either the vehicle (PBS, 30 μL, i.pl.) or 1.5 mg/30 μL MSU (i.pl.). The paw volume was evaluated at the same time points as previously described. The percentage of edema inhibition by the compounds was calculated discounting the paw volume of mice that received oral or intraperitoneal vehicle and PBS intraplantar (VEH + VEH).

### Assessment of Paw Tissue Peroxidase Activity and Cytokine Levels

After the assessment of paw edema was completed, the mice were immediately anesthetized by inhalation of 2% isoflurane mixture in oxygen (O_2_). Subsequently, the surface of the hind paws was collected for the measurement of total peroxidase activity, IL-6, and IL-1β. These tissue samples were then frozen using dry ice and stored at −80 °C until further analysis. The frozen tissues were later pulverized into a fine powder using liquid nitrogen and homogenized in a phosphate buffer (100 mM, pH 7.4). For the analysis of total peroxidase activity, a portion of this homogenate was mixed with hexadecyltrimethylammonium (1% HTAB, pH 6.0), vigorously vortexed, and sonicated. The resulting mixture was then centrifuged at 10,000*g* for 10 min, at 4 °C, and heated at 60 °C for 2 h. The sample of each supernatant was incubated with a solution containing 50 mM phosphate buffer, 0.164 mg/mL of *o*-dianisidine dihydrochloride, and H_2_O_2_ (0.0005%, pH 6.0). The colorimetric reaction was monitored using spectrophotometry (optical density, O.D; 460 nm; Spectra Max plus 384, Sunnyvale, CA, USA), and expressed as Unit (U) per mg of protein. Each unit represents the capacity to degrade 1 μmol of H_2_O_2_ per minute.^81^

The remaining part of the homogenate was treated with a solution of protease/phosphatase inhibitors (1 mM phenylmethylsulfonyl fluoride, 10 μg/mL leupeptin, and 10 μg/mL trypsin inhibitor) and centrifuged at 10,000*g* (10 min at 4 °C). The obtained supernatants were subjected to 1:2 dilution in PBS and subsequently used for the assessment of IL-1β and IL-6 using murine enzyme-linked immunosorbent assay kits, specifically a sandwich ELISA technique, following manufacturer’s instructions (BioLegend, CA, USA). The protein content within the samples was determined by using the Bradford method (1976). The results were quantified and expressed in pg/mg of protein (Bio-Rad, Hercules, CA, USA).^76^

### Statistical Analysis

Group differences were analyzed through one-way analysis of variance (ANOVA) or two-way ANOVA with repeated measures, followed by Bonferroni’s multiple comparison posthoc test. All results are expressed as mean ± standard error of mean (SEM), with a significance level set at *P* < 0.05. The analyses were carried out using GraphPad Prism version 6.0 (GraphPad Software, San Diego, USA).

## Supporting Information Available

Alignments of MPO crystal structures; molecular docking parameters of MPO inhibitors from AutoDock; UV/vis and fluorescence scans of RL6 and RL7; independent replicates for NETosis assay; evaluation of RL6 off-target effect; evaluation of cytotoxicity by XTT; and purity confirmation of the compounds that were active against MPO chlorinating and peroxidatic activities m (PDF)
Molecular formula strings (CSV)

## Abbreviations

DFT: density functional theory
DMSO: dimethyl sulfoxide
EPO: eosinophil peroxidase
GBD: global burden of disease
HOCl: hypochlorous acid
HOMA: harmonic oscillator model of aromaticity
HTS: high-throughput screening
IL-1β: interleukin 1β
IL-6: interleukin 6
LPO: lactoperoxidase
MPO: myeloperoxidase
MSU: monosodium urate
NETs: neutrophil extracellular traps
PTU: propylthiouracil
TPO: thyroid peroxidase
TPSA: topological surface area
