# Supplementary material for: Targeting Myeloperoxidase Ameliorates Gouty Arthritis: A Virtual Screening Success Story
Source: J Med Chem. 2024 Jul 11;67(14):12012–32. doi: 10.1021/acs.jmedchem.4c00721 (PMC11284790; doi:10.1021/acs.jmedchem.4c00721)
Supplement: Supplementary file 1 — jm4c00721_si_001.pdf [file jm4c00721_si_001.pdf]

Supporting Information

Targeting Myeloperoxidase Ameliorates Gouty Arthritis: A Virtual Screening Success Story

Isaac de A. Matos[1*], Jorge L. Dallazen[2], Lorenna R. Reis[1], Luiz Felipe Souza[1], Regina C. Bevevino[1], Rafael D. de Moura[1], Graziella E. Ronsein[1], Nicolas Carlos Hoch[1], Nivan Bezerra da Costa Júnior[3], Soraia Kátia P. Costa[2], Flavia C. Meotti[1*]

[1]*Department of Biochemistry, Institute of Chemistry, University of São Paulo, São Paulo, 05508-000, Brazil*

[2]*Department of Pharmacology, Institute of Biological Sciences, University of São Paulo, São Paulo* 05508-000, *Brazil*

[3]*Department of Chemistry, Federal University of Sergipe, Sergipe 49100-000, Brazil*

*To whom correspondence should be addressed:

Flavia Carla Meotti or Isaac de Araújo Matos

Department of Biochemistry, Institute of Chemistry, University of São Paulo, São Paulo, Brazil

Av. Prof Lineu Prestes, 748. Office 1004

Postal code 05508-000, Tel.: +55 11 3091-1763

Email: flaviam@iq.usp.br, isaacbioquim@usp.br

Contents

Figure S1. Alignments of MPO crystal structures..S3
Table S1. Molecular docking parameters of MPO inhibitors from AutoDock.............S4
Figure S2. UV/Vis and fluorescence scans of RL6 and RL7…………………………..S6
Figure S3. Independent replicates for NETosis assay..S7
Figure S4. Evaluation of RL6 off-target effect..S8
Figure S5. Evaluation of cytotoxicity by XTT..S9
Figure S6. Purity confirmation of the compounds that were active against MPO chlorinating and peroxidatic activities..S10
Molecular Formula Strings File (csv format separately)

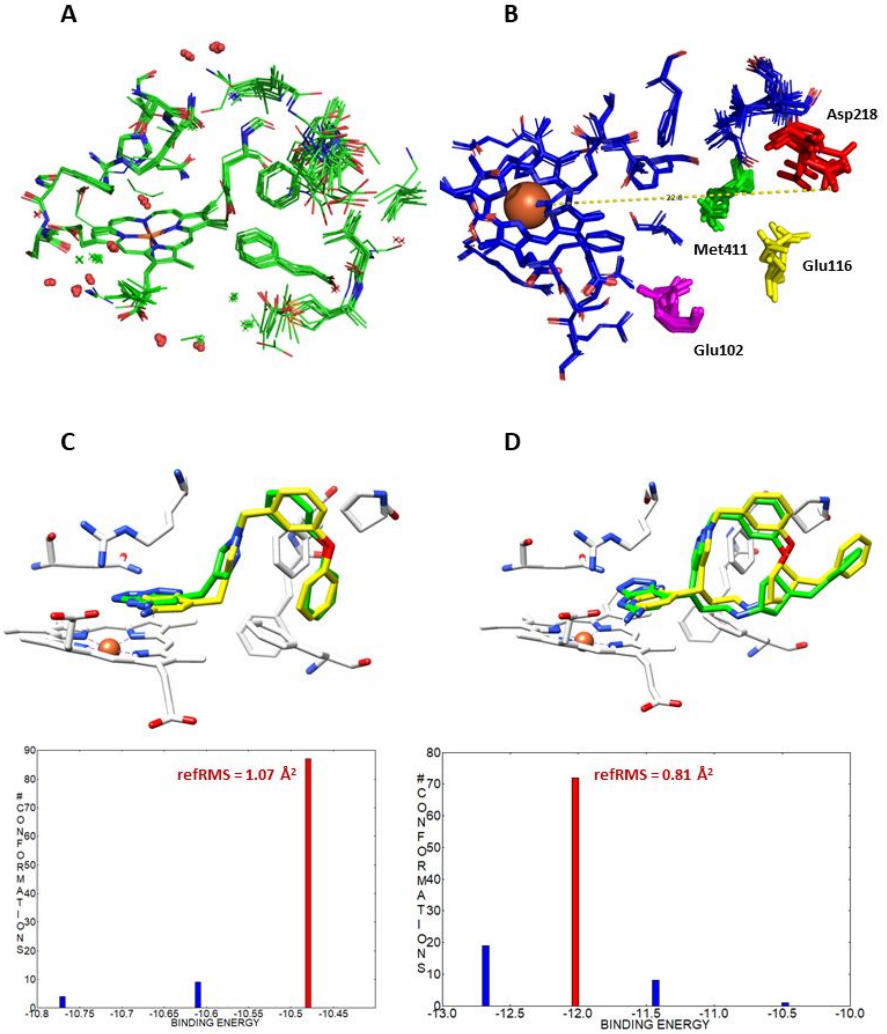

Figure S1. Alignments of MPO crystal structures PDB 5QJ2, 5QJ3, 6WXZ, 6WY0, 6WY5, 6WY7, 6WYD, 7LAE, 7LAG, 7LAL and 7LAN. (A) Conserved water molecules are shown as red spheres. (B) Rigid residues are shown in blue lines and the flexible ones in green (Met411), red (Asp218), yellow (Glu116) and magenta (Glu102). Distance between iron atom and Asp218 is shown by the yellow dot line. Cross-docking of the ligands present in the active site of PDB 7LAG (C) and 7LAN (D) with the PDB1CXP structure. Pose are shown in green and crystallographic conformation in yellow. The respective conformational histograms are shown underneath the structures. The most populous cluster is in red with its respective refRMS value.

Table S1. Molecular docking parameters of MPO inhibitors from AutoDock

Structure	In house	$\Delta G_{binding}$	K_i (nM)	Ligand Efficiency	Desolvation energy	Electrostatic energy	Hydrogen bonds	Histogram
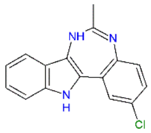	RL1	-7.81	1,900	-0.39	-7.62	-0.19	2	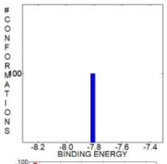
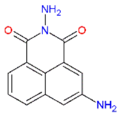	RL6	-7.84	1,810	-0.46	-8.38	-0.05	1	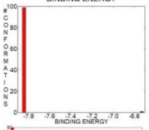
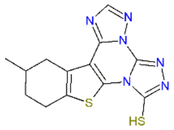	(S) RL9	-7.6	2700	-0.36	-8.12	-0.67	2	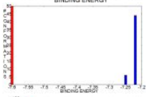
	(R) RL9	-8.24	0.906	-0.39	-8.68	-0.75	2	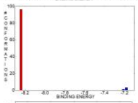
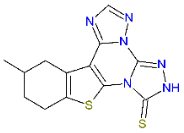	(S) RL9	-7.60	2690	-0.36	-7.55	-0.95	0	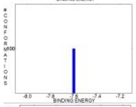
	(R) RL9	-7.63	2570	-0.36	-7.57	-0.95	0	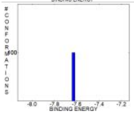
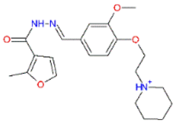	RL15	-8.45	0.638	-0.3	-9.3	-1.54	3	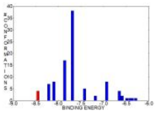
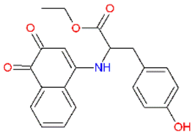	(S) RL16	-8.21	0.963	-0.3	-10.48	-0.11	3	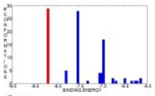
	(R) RL16	-7.73	2160	-0.29	-10.04	-0.08	2	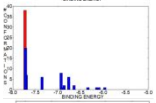
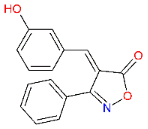	RL17	-7.57	2,832	-0.38	-8.43	-0.03	2	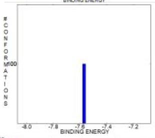
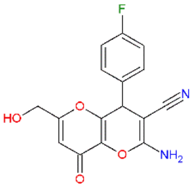	(S) RL19	-7.23	5,000	-0.31	-8.4	-0.02	3	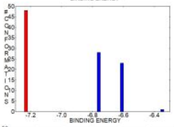
	(R) RL19	-7.43	3590	-0.32	-8.47	-0.15	3	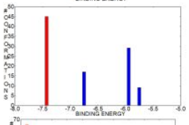
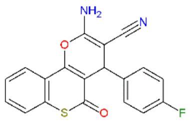	(S) RL20	-7.78	1980	-0.31	-8.27	-0.11	2	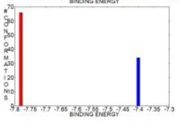
	(R) RL20	-8.84	0.333	-0.35	-9.23	-0.2	1	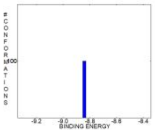

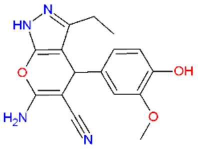	(S) RL23	-7.81	1,890	-0.34	-8.95	-0.35	4	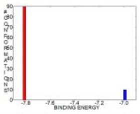
	(R) RL23	-7.39	3840	-0.32	-8.68	-0.21	3	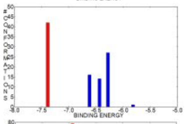
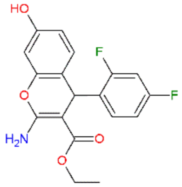	(S) RL24	-6.94	8160	-0.28	-8.58	-0.15	2	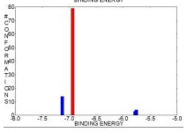
	(R) RL24	-8.19	0.999	-0.33	-9.84	-0.14	4	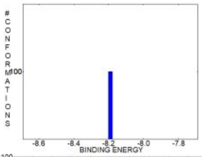
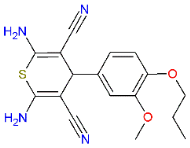	RL26	-7.37	3,930	-0.31	-9.1	-0.36	2	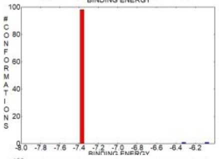
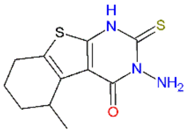	(S) RL27	-7.09	6350	-0.42	-7.42	0.03	1	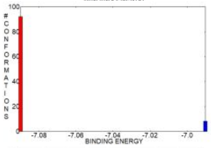
	(R) RL27	-6.68	12670	-0.39	-6.86	-0.12	0	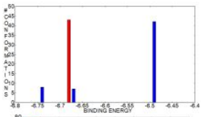
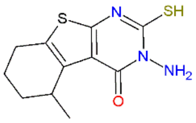	(S) RL27	-7.62	2590	-0.45	-8.08	-0.13	1	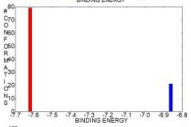
	(R) RL27	-6.94	8170	-0.41	-7.48	-0.06	0	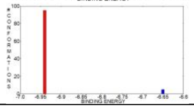

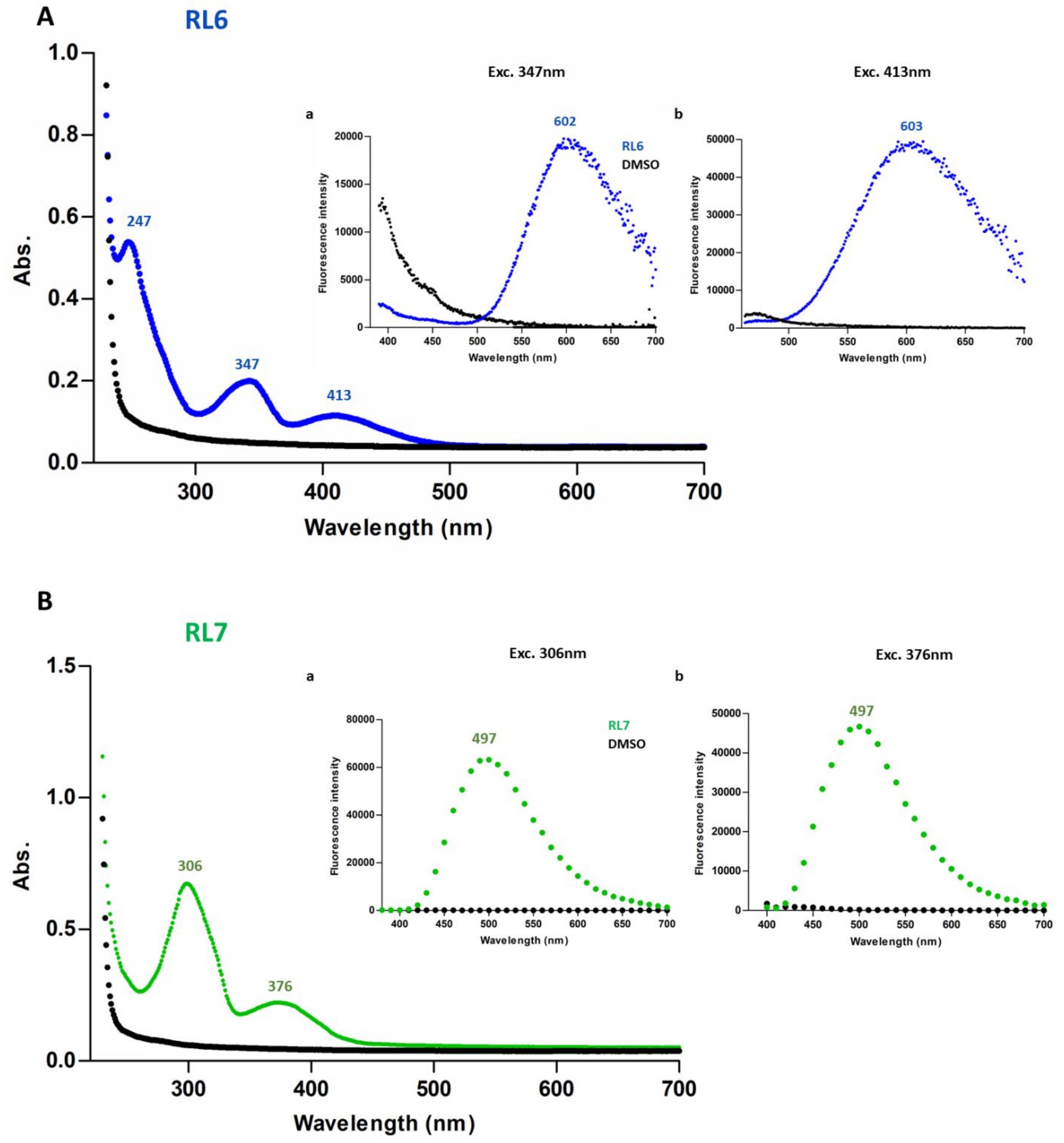

Figure S2. UV/Vis and fluorescence scans of RL6 and RL7. (A) RL6 absorbance spectrum (left) and its respective fluorescence emission when excited at 347 and 413 nm (right). **(B)** RL7 absorbance spectrum and its respective fluorescence emission spectrum excited at 306 and 376 nm. Spectra were obtained at 20 µM in DMSO with a gain 100 to RL7 and 150 to RL6 using a microplate reader.

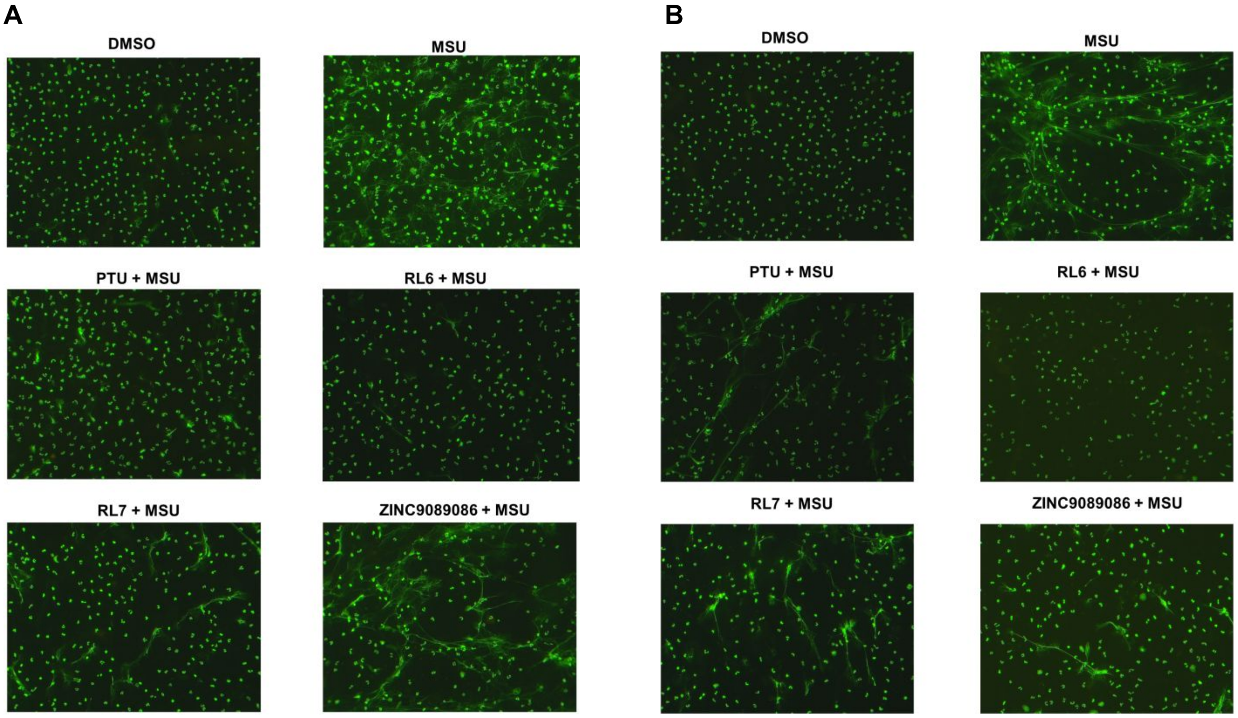

Figure S3. Independent replicates for NETosis assay, n=2 **(A)** and n=3 **(B)**. Adhered neutrophils were covered with RPMI medium containing 20 μM compounds and MSU (250 μg/mL). After 90 min incubation cells were fixed and kept at 4ºC overnight. Cells were then washed three-fold with tris-HCl buffer (20 mM, pH 7.4) and DNA was stained by 500 μL sytox green (500 nM) for 30 min. After coverslips mounted and fixed, the fluorescence images were acquired by the fluorescence microscope (λ_{ex} = 450 - 490 nm, λ_{em} = above 515 nm).

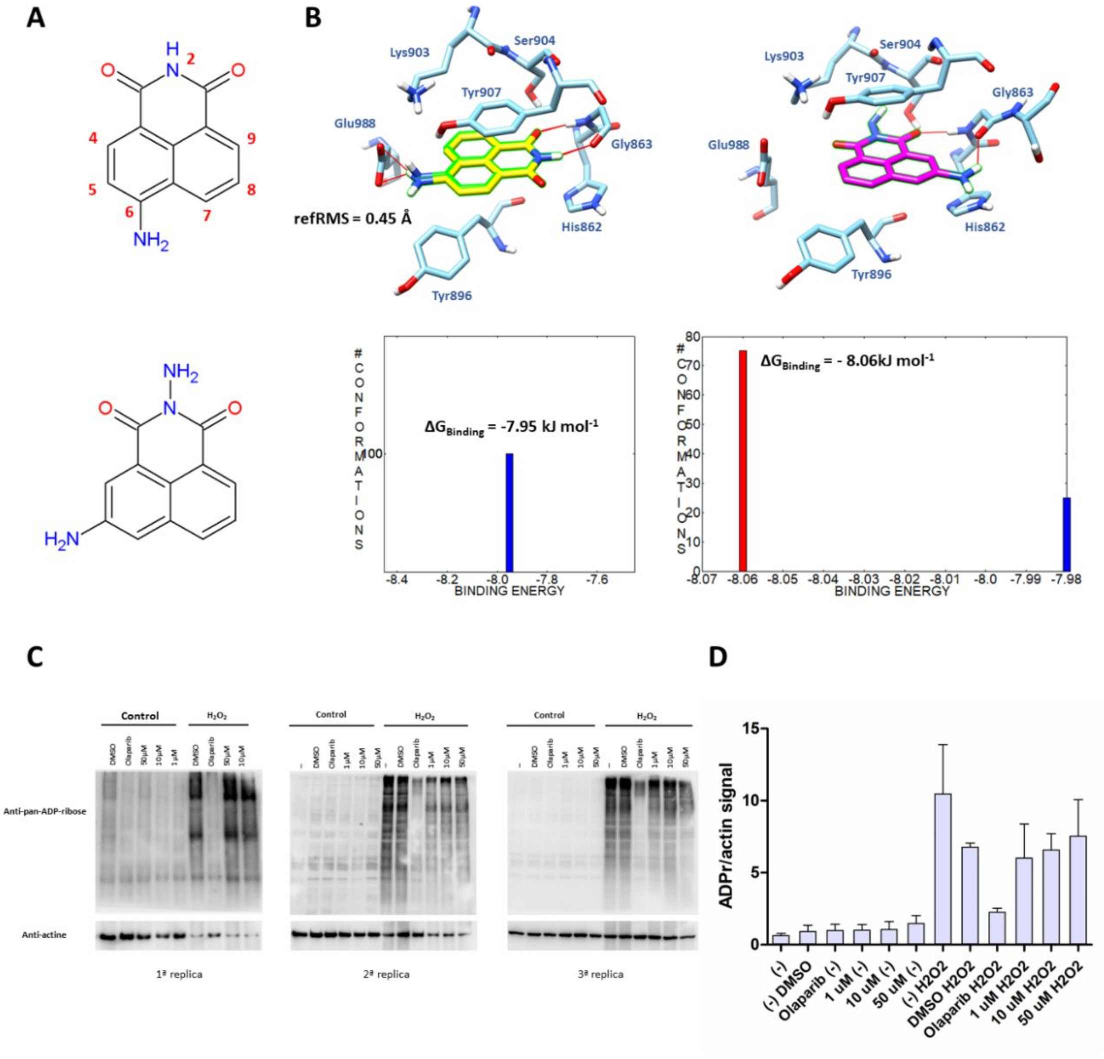

Figure S4. Evaluation of RL6 off-target effect. (A) Molecular structure of the PARP1 inhibitor 4ANI (top) and RL6 (bottom) showing their structural similarity **(B)** Left, 4ANI redocking into the PARP1 active site by experimental crystallography (green) and redocking (yellow). Molecular docking of RL6 (magenta) at the right. The respective histograms and binding energies are shown below. For RL6, the conformation with the best binding energy of the most populous cluster was selected. For molecular docking studies with PARP1, the PDB 2PAX structure was selected, the hydrogens were added and water molecules were maintained during simulation. All simulations were performed using AutoDock 4.2.3 in a box of 40x, 40y,40z dimensions and centered in the coordinates 40.852x, 22.791y, 21.55z. The Lamarckian genetic algorithm was selected and it was run 100 times. Other parameters were kept as default. (**C**) ADP-ribosylation in RPE1-hTERT cells was triggered by H_2O_2 (600 μM) for 10 min. (**D**) Gel densitometry quantified by ImageJ software, n=3. A concentration of 0.1% DMSO was used.

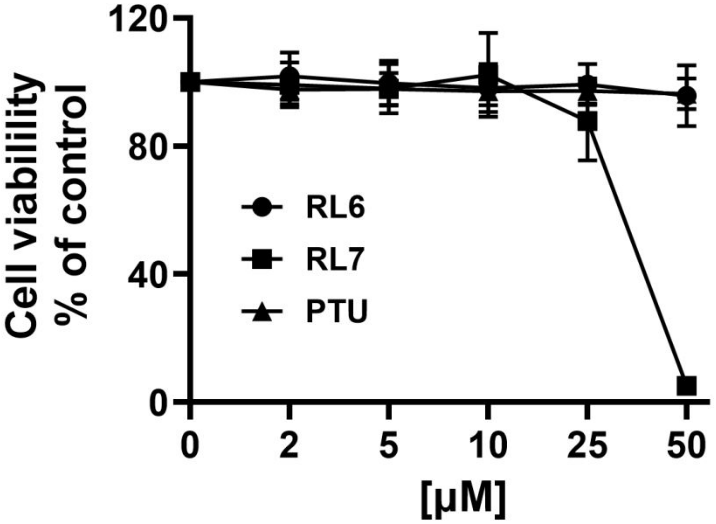

Figure S5. Evaluation of cytotoxicity by XTT. hTERT-RPE1 cells were seeded in 24-well plates at 2 × 10^4 cells per well. 24h later, cells were treated with RL6, RL7 or PTU (2, 5, 10, 25, 50 μM) in culture medium (DMEM/F-12 with HEPES and 10% FBS). Viable cells were assessed 72h after treatments by measuring absorbance of the supernatant at 450 and 750 nm and are presented as percentage of control (0.42% DMSO). Data are mean ± SEM of three independent experiments.

RL1

SPECS and BioSPECS B.V., The Netherlands Page 156
File:9910134227041 ID:AH-262/34227041 Description:C16H12ClN3
Vial:2:24 Date:26-Aug-2000 Time:11:33:09

Printed: Wed Sep 06 12:44:13 2000

Sample Report (continued):

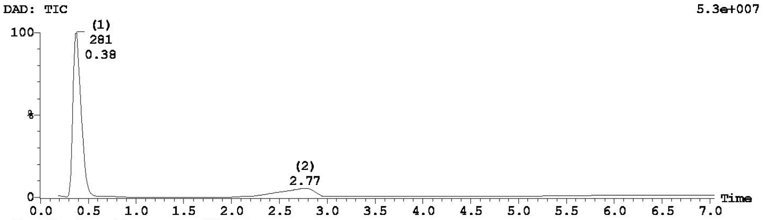

Peak Number	Time	Area %Total
1	0.38	84.22
2	2.77	15.78

Peak ID	Time	Mass Found	BPM
1	0.38	281.07	282.1

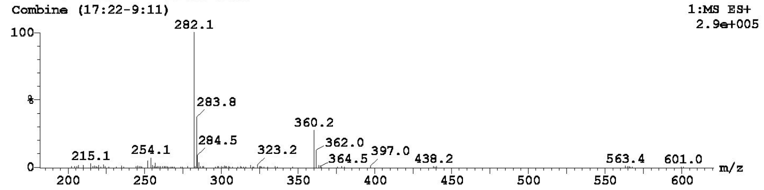

Peak ID	Time	Mass Found	BPM
2	2.77		300.2

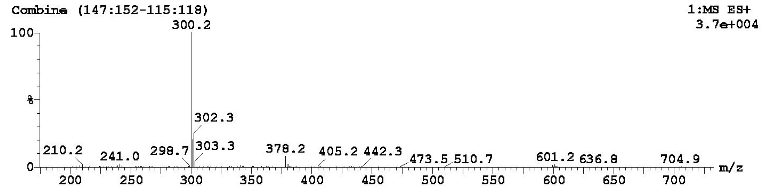

RL6

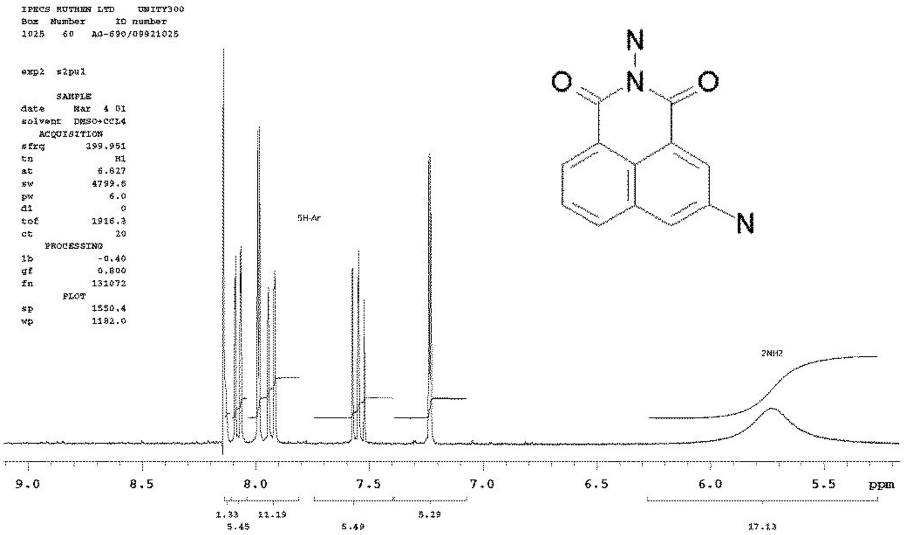

RL7

Specs
File:5000142860464 | ID:AM-807/42860464 | Description:C19H17N5OS | Page 68
Instrument:LC/MS A | Vial:1:68 | Date:07-Oct-2004

Printed: Fri Oct 08 16:04:40 2004

Sample Report (continued):

Diode Array Detector 240 - 320 nm: TIC 1.2e+008

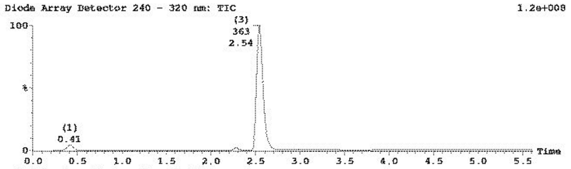

Peak Number	Time	Area %Total
1	0.41	4.16
2	2.23	1.49
3	2.54	94.35

Peak ID	Time	Mass Found	BPM
2	2.28		226

Combine (120:125-115:117) 1:MS ES+ 1.2e+003

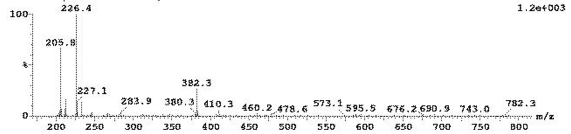

Peak ID	Time	Mass Found	BPM
3	2.54	363.12	364

Combine (135:140-127:130) 1:MS ES+ 3.9e+004

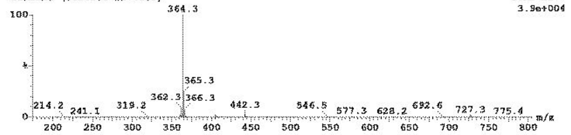

RL9

Specs
File:9900142926896 | ID:AL-281/42926896 | Description:C13H12N6S2 | Page 150
Instrument:LC/MS A | Vial:2:18 | Date:05-Feb-2004

Printed: Thu Feb 05 15:13:09 2004

Sample Report (continued):

Diode Array Detector 240 - 320 nm: TIC 8.8e+007

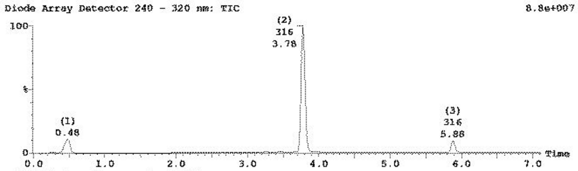

Peak Number	Time	Area %Total
1	0.48	12.37
2	3.78	80.02
3	5.88	7.61

Peak ID	Time	Mass Found	BPM
1	0.48		215

Combine (22:27-12:15) 1:MS ES+ 2.1e+003

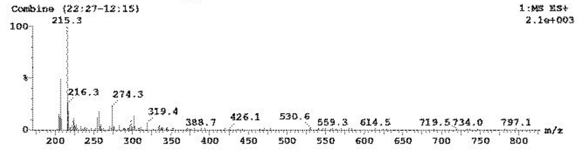

Peak ID	Time	Mass Found	BPM
2	3.78	316.06	317

Combine (202:207-191:194) 1:MS ES+ 7.7e+003

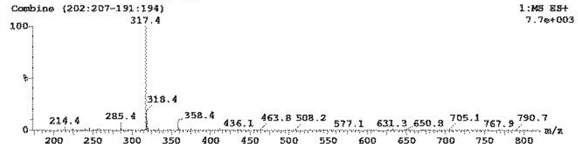

RL17

SPECS and BioSPECS B.V., The Netherlands Page 30
File:3910113582003 ID:AH-487/13582003 Description:C16H11NO3
Vial:1:30 Date:08-Mar-2000 Time:21:11:13

Printed: Tue Apr 24 11:00:52 2001

Sample Report (continued):

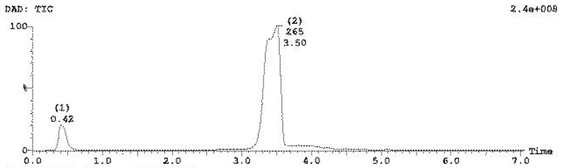

Peak Number	Time	Area %Total
1	0.42	10.88
2	3.50	89.12

Peak ID	Time	Mass Found	BPM
1	0.42		257.0

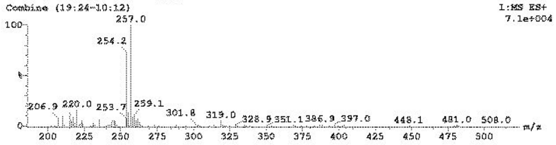

Peak ID	Time	Mass Found	BPM
2	3.50	265.07	266.1

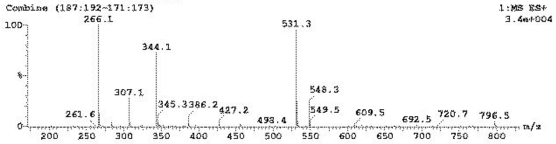

RL19

Specs Page 118
File:5000141931995 ID:AM-807/41931995 Description:C16H11FN2O4
Instrument:LC/MS A Vial:1:118 Date:30-Sep-2004

Printed: Thu Sep 30 16:33:40 2004

Sample Report (continued):

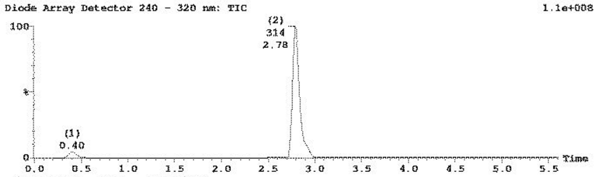

Peak Number	Time	Area %Total
1	0.40	4.33
2	2.78	95.67

Peak ID	Time	Mass Found	BPM
1	0.40		257

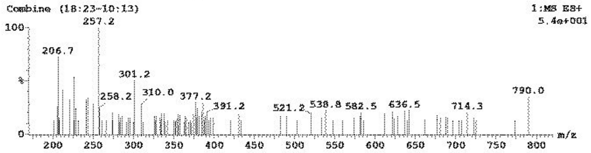

Peak ID	Time	Mass Found	BPM
2	2.78	314.07	315

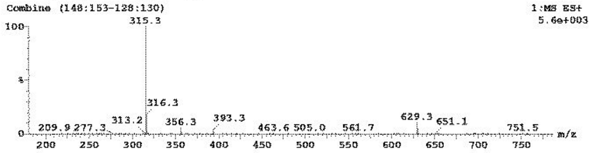

Figure S6. Purity confirmation of the compounds that were active against MPO chlorinating and peroxidatic activities. LC/UV-Vis, LC/MS and NMR data were provided by the manufacturer.
